# Targeted isolation of TolC-dependent phages reveals dual strategies for combating multidrug-resistant avian *Escherichia coli*: from evolutionary trade-offs to antibiotic synergy

**DOI:** 10.3389/fmicb.2026.1868543

**Published:** 2026-07-14

**Authors:** Xinwei Luo, Yiting Wu, Yue Ming, Jian Wang, Zhenbao Ma, Anding Zhang, Shengdi Hu

**Affiliations:** 1 Animal Husbandry and Fisheries Research Center of Guangdong Haid Group Co., Ltd., Guangzhou, China; 2 Key Laboratory of Preventive Veterinary Medicine in Hubei Province, The Cooperative Innovation Center for Sustainable Pig Production, Wuhan, Hubei, China; 3 Key Laboratory of Microecological Resources and Utilization in Breeding Industry, Ministry of Agriculture and Rural Affairs, Guangdong Haid Group Co., Ltd., Guangzhou, China

**Keywords:** avian pathogenic *Escherichia coli*, bacteriophage therapy, evolutionary trade-off, multidrug resistance, phage-antibiotic synergy, poultry production, TolC protein

## Abstract

The escalating crisis of antimicrobial resistance (AMR) in poultry production necessitates innovative therapeutic approaches beyond conventional antibiotics. The outer membrane protein TolC, an essential component of the AcrAB-TolC multidrug efflux system, is consistently overexpressed in multidrug-resistant (MDR) avian pathogenic *Escherichia coli*, rendering it an attractive target for phage-based interventions. Here, we describe the development of a sequential positive–negative selection strategy designed specifically to isolate TolC-dependent bacteriophages from poultry farm environments without conventional liquid enrichment, thereby preserving natural phage diversity. Using this approach, we successfully isolated two novel TolC-dependent bacteriophages, PTolC-28 and PTolC-69, demonstrating their ability to combat MDR *E. coli* through two distinct mechanisms. Among the five MDR isolates susceptible to PTolC-28, one strain (GDW21C03) displayed a pronounced, strain-specific evolutionary trade-off upon developing resistance: despite maintaining an intact coding sequence, *tolC* mRNA expression decreased by over 60%, resulting in collateral resensitization to multiple antibiotic classes. The most pronounced reductions in minimum inhibitory concentrations (MICs) occurred for fluoroquinolones (~5.3-fold), tetracyclines, and aminoglycosides, all substrates of the TolC-dependent efflux system(s). Conversely, PTolC-69 did not induce antibiotic resistance reversal but exhibited robust phage-antibiotic synergy (PAS) with doxycycline (DOX) and florfenicol (FLR) (both substrates of the AcrAB-TolC efflux system), reducing the required antibiotic dosage by 8-fold *in vitro*. Importantly, this synergistic effect was confirmed *in vivo* using a chick infection model, where combined phage-antibiotic therapy decreased bacterial loads in lung and spleen tissues by nearly two orders of magnitude compared to either treatment alone. Collectively, these findings provide proof-of-concept evidence for an evolution-informed, dual-mechanism phage-based strategy to address antibiotic resistance in poultry production.

## Graphical abstract

**Figure:**
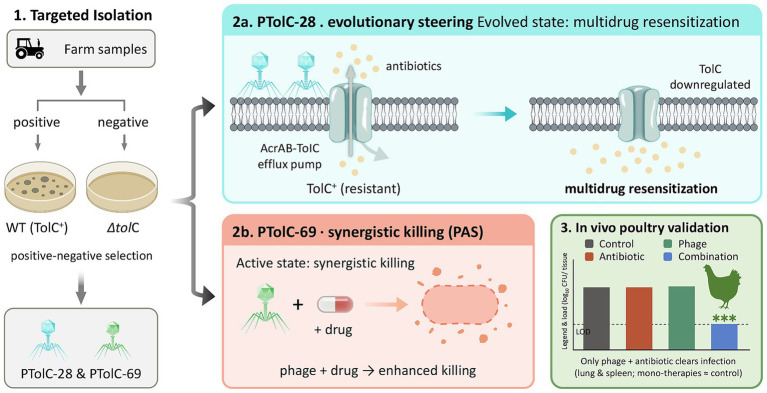

## Introduction

Antimicrobial resistance (AMR) has escalated from a future threat to a present-day global crisis, directly causing an estimated 1.14 million deaths in 2021, with projections suggesting direct mortality could reach 1.91 million per year by 2050 (Naghavi et al., 2024). The World Health Organization currently ranks AMR among the top 10 global public health threats due to its significant implications for mortality, morbidity, and economic stability (World Health Organization, 2025). Animal agriculture significantly contributes to this crisis, accounting for more than 70% of global antimicrobial consumption. In food animal production, antimicrobials are frequently employed not only for therapeutic purposes but also prophylactically and as growth promoters within intensive farming systems (Van Boeckel et al., 2015; Mulchandani et al., 2023). The poultry industry, in particular, has emerged as a major reservoir for the emergence and dissemination of multidrug-resistant (MDR) pathogens, driven by the scale and intensive nature of poultry farming practices (Mulchandani et al., 2023). Among these, *Escherichia coli* is a primary etiological agent of colibacillosis, causing substantial economic losses worldwide (Kathayat et al., 2021; Acharya and Barsila, 2025). The rising prevalence of MDR *E. coli* isolates from diseased poultry severely undermines conventional antibiotic treatments, highlighting the urgent demand for innovative and sustainable therapeutic alternatives (Lúcio et al., 2025; Naidoo and Zishiri, 2025).

A primary mechanism underlying MDR in Gram-negative bacteria, including *E. coli*, involves the overexpression of efflux pump systems, such as the AcrAB-TolC complex (Nikaido and Zgurskaya, 2001; Jang, 2023). These molecular pumps actively expel diverse classes of antibiotics from bacterial cells, compromising antibiotic efficacy (Venter et al., 2015). Central to these systems is the outer membrane protein TolC, a universal exit duct for multiple efflux pumps, crucially contributing to the MDR phenotype (Nikaido and Zgurskaya, 2001; Koronakis et al., 2004). While efflux pumps confer intrinsic resistance, regulatory mutations leading to their overexpression represent a major pathway toward high-level, clinically significant MDR (Pagès and Amaral, 2009; Pourahmad Jaktaji and Zargampoor, 2017). Indeed, elevated *tolC* expression correlates directly with heightened MDR in clinical *E. coli* isolates (Viveiros et al., 2007). Given this pivotal role, TolC serves not only as a marker for MDR but also as a promising yet challenging target for novel antibacterial strategies (Nikaido and Pagès, 2012; Burmeister et al., 2020).

Bacteriophage therapy has reemerged as a promising alternative against MDR bacteria due to its high specificity and inherent self-replicating capability (Lin et al., 2017; Furfaro et al., 2018; Marongiu et al., 2022; Albarella et al., 2025). However, the rapid emergence of bacterial resistance, frequently via mutations in the surface receptors utilized by phages for attachment, presents a significant challenge to its effectiveness (Luria and Delbrück, 1943; Bertozzi Silva et al., 2016; Furfaro et al., 2018; Zhao et al., 2024; Gomaa et al., 2025; Li et al., 2025). Consequently, more strategic approaches have emerged, including targeting bacterial structures essential for survival or virulence (Miele et al., 2023). Notably, some phages have evolved mechanisms to exploit these critical bacterial components, such as efflux pump proteins, as receptors (Burmeister et al., 2020). In particular, the outer membrane protein TolC experiences intense selective pressure, rendering it a highly evolvable target under phage attack (Tamer et al., 2021). This adaptability underpins a significant evolutionary trade-off: bacteria either remain susceptible to phage infection or regain sensitivity to multiple antibiotics through mutations or downregulation of *tolC*, which prevents phage attachment (Burmeister et al., 2020). This principle, termed “phage steering,” involves deploying phages whose receptors are antibiotic resistance determinants, making phage resistance impose a cost in antibiotic sensitivity (Pál et al., 2015). It was first demonstrated in *P. aeruginosa* with phage OMKO1 targeting the OprM efflux channel (Chan et al., 2016), applied clinically (Chan et al., 2018), and further characterized evolutionarily (Gurney et al., 2020; Kortright et al., 2022). A similar mechanism has since been shown in *Burkholderia* (Ruest et al., 2023). This strategy is mechanistically distinct from phage-antibiotic synergy (PAS), where sublethal antibiotic concentrations enhance phage killing without necessarily invoking evolutionary trade-offs (Olawade et al., 2024; Supina and Dennis, 2025; Lauman et al., 2026).

However, the general applicability of this evolutionary trade-off cannot be assumed, as its effectiveness depends critically on whether phage-resistance and antibiotic-resistance functions of TolC can be evolutionarily decoupled. Fundamental research on this evolutionary interaction, particularly using the model phage TLS, has yielded essential insights into the underlying molecular dynamics. A detailed mapping of the TolC mutational landscape revealed that resistance to TLS could arise via mutations in its extracellular loops, often without impairing antibiotic efflux activity (Tamer et al., 2021). The discovery that these two functions can be evolutionarily separated thus raises an important question regarding the generalizability of this model. Whether this efflux-preserving escape mechanism is broadly applicable or whether phages isolated from diverse natural environments, particularly agricultural contexts that foster MDR *E. coli* emergence, impose more stringent evolutionary constraints remains largely unexplored.

In this study, we developed a sequential positive–negative selection strategy to isolate TolC-dependent bacteriophages directly from poultry farm environments and assessed their therapeutic potential against MDR avian pathogenic *E. coli* strains originating from the same ecological niche. Specifically, we sought to determine whether these phages could impose therapeutically meaningful selective pressures on MDR *E. coli*, identify the mechanisms involved, and elucidate the host-specific conditions under which such effects occur. To achieve this, we systematically characterized the lytic spectra, resistance-reversal capacities, and antibiotic synergy profiles of the newly isolated phages against a panel of poultry-derived MDR isolates, with the ultimate goal of defining the therapeutic modalities achievable by targeting TolC.

## Materials and methods

### Bacterial strain isolation, identification, and molecular typing

Ten pathogenic *E. coli* strains were isolated from heart and liver tissues of deceased chickens obtained from poultry farms in Guangdong Province, China. Tissue samples were streaked onto Eosin Methylene Blue (EMB) agar and incubated at 37 °C for 24 h. Colonies exhibiting the characteristic green metallic sheen were selected and purified through two subsequent rounds of streaking onto fresh EMB agar. Pure isolates were preserved in LB broth containing 40% glycerol at −80 °C. Species identity was confirmed by sequencing the 16S rRNA gene, and sequences were compared with those in the NCBI database using BLAST.

Multilocus sequence typing (MLST) was conducted by amplifying seven housekeeping genes (*adk*, *fumC*, *gyrB*, *icd*, *mdh*, *purA*, and *recA*) via PCR. The amplicons were sequenced, and the resulting sequences were submitted to the *E. coli* MLST database (PubMLST) to determine allelic profiles and assign sequence types (STs).

### Quantitative real-time PCR (RT-qPCR) analysis

The mRNA expression levels of the *tolC* gene were quantified using one-step RT-qPCR. *E. coli* strains were cultured overnight in Luria-Bertani (LB) broth at 37 °C with shaking at 180 rpm, then diluted 1:100 into fresh LB medium and grown to mid-logarithmic phase (OD_600_ ≈ 0.6) under antibiotic-free conditions. Total RNA was extracted using the Bacteria RNA Extraction Kit (Vazyme Biotech Co., Ltd., Nanjing, China) according to the manufacturer’s instructions. RNA concentration and purity (A_260_/A_280_) were determined using a NanoDrop spectrophotometer, and RNA integrity was verified by agarose gel electrophoresis. One-step RT-qPCR was performed with the AccurStart U + One Step RT-qPCR SuperMix (Vazyme Biotech Co., Ltd.) on a qTOWER^3^ Real-Time PCR System (Analytik Jena GmbH, Jena, Germany), using equal RNA input (0.2 ng per reaction). Cycling conditions followed the manufacturer’s recommendations. The 16S rRNA gene served as the endogenous control for data normalization. Primer and TaqMan probe sequences for *tolC* and 16S rRNA are provided in Supplementary Table S1. Experiments were performed in three independent biological replicates, each analyzed in technical triplicate. Relative *tolC* expression was calculated using the 2^−ΔΔCt^ method (Livak and Schmittgen, 2001), with *E. coli* MG1655 as the calibrator (relative expression = 1). No-template (NTC) and no-reverse-transcriptase (no-RT) controls were included in each run.

### Construction of the *Escherichia coli ΔtolC* mutant

The *tolC* deletion mutant was constructed using the CRISPR-Cas9 system as previously described (Jiang et al., 2015; Li et al., 2021). Briefly, a specific guide RNA targeting the *tolC* gene was cloned into the pEcgRNA vector. The resulting plasmid, together with a homologous recombination donor DNA fragment, was co-electroporated into *E. coli* MG1655 harboring the pEcCas plasmid. Successful gene deletion was confirmed by colony PCR and sequencing. The pEcgRNA and pEcCas plasmids were subsequently cured from the verified mutant strain.

### Isolation of phage-resistant mutants

Phage-resistant mutants were obtained through co-culture selection. Each of the five parental *E. coli* strains susceptible to PTolC-28 was grown in LB broth at 37 °C and 200 rpm to mid-logarithmic phase (OD_600_ ≈ 0.5; ~1–2 × 10^9^ CFU/mL). Cultures were then co-inoculated with TolC-dependent phage into fresh LB broth supplemented with 5 mM CaCl_2_ and 5 mM MgCl_2_, achieving a final bacterial concentration of approximately 1 × 10^5^ CFU/mL and a phage titer of ≥ 1 × 10^9^ PFU/mL (MOI ≥ 10,000). Cultures were incubated at 37 °C, 200 rpm, and monitored until OD_600_ values recovered to ≥ 0.5 (typically within 12–24 h). Cultures failing to recover within 48 h were discarded. Selection was performed in three independent biological replicates for each parental-phage pair.

Resistant clones were identified through a two-step confirmation procedure. First, recovered co-cultures were mixed with molten top agar and overlaid onto LB agar to form bacterial lawns, alongside control lawns prepared from untreated parental strains. A high-titer phage lysate (10 μL, 1 × 10^9^ PFU/mL) was spotted onto both lawns, followed by incubation overnight at 37 °C. Regions showing reduced or absent lysis on co-culture lawns, compared to clear lysis zones on parental controls, were sampled and streaked onto EMB agar for single-colony purification. Single colonies were then inoculated into LB broth, cultured to mid-logarithmic phase, and validated by a second spot assay conducted in parallel with untreated parental strains. Isolates demonstrating complete resistance, characterized by the absence of a lysis zone, in contrast to clear lysis observed in parental controls, were confirmed as phage-resistant mutants. Confirmed mutants were passaged daily for five consecutive transfers in phage-free LB medium, and resistance was reconfirmed via spot assay to exclude transient phenotypes.

### Targeted isolation of TolC-dependent bacteriophages

A sequential positive–negative selection strategy was established for the targeted isolation of TolC-dependent bacteriophages, deliberately omitting conventional liquid enrichment to maintain natural viral diversity. Environmental samples, such as chicken feces, were processed into bacteria-free crude lysates through centrifugation followed by filtration through a 0.22 μm membrane.

In the positive selection step, the filtrate was passed through a PES vacuum filtration unit (0.45 μm + 0.2 μm, Cobetter) precoated with a lawn of wild-type *E. coli* MG1655. The filter membrane was then aseptically transferred to buffer solution and vortexed to resuspend host cells along with the adsorbed phages.

In the negative selection step, the recovered phage suspension was pre-incubated with *E. coli* MG1655Δ*tolC*. Following incubation, bacterial cells and bound phages were removed by centrifugation. This depletion procedure was repeated three to six times.

The resulting TolC-enriched phage suspension was then plated using the double-layer agar method onto an indicator lawn of *E. coli* MG1655 and incubated overnight at 37 °C. Emerging plaques were picked and spotted simultaneously onto lawns of wild-type MG1655 and MG1655Δ*tolC*. Candidates exhibiting markedly reduced plaque formation on the Δ*tolC* lawn were selected and purified through three successive rounds of single-plaque isolation. TolC-dependence was reconfirmed by differential plating of the final purified isolates (Burmeister et al., 2020; Maffei et al., 2021; Burmeister et al., 2023).

### Phage adsorption assay

Adsorption of each phage to wild-type *E. coli* MG1655 and the MG1655Δ*tolC* mutant was evaluated at 37 °C. Mid-logarithmic host cells (OD_600_ ≈ 0.5) in LB supplemented with 5 mM CaCl_2_ and 5 mM MgCl_2_ were infected at an MOI of 0.01. At 0, 2, 4, 6, 8, and 10 min, aliquots were filtered through a 0.22 μm filter to remove cells and cell-bound phages. The titer of free phages in the filtrate was determined by the double-layer agar method.

### One-step growth curve

One-step growth curves of PTolC-28 and PTolC-69 were determined using wild-type *E. coli* MG1655. Mid-logarithmic-phase cells were infected at an MOI of 0.01 and allowed to adsorb at 37 °C for 5 min. Unadsorbed phages were then removed by centrifugation (8,000 rpm, 5 min, 4 °C). The cell pellet was resuspended in fresh pre-warmed LB and incubated at 37 °C with shaking. Aliquots were collected at regular intervals (every 10 min for 90 min for PTolC-28 and every 5 min for 45 min for PTolC-69). At each time point, one aliquot was directly titrated to quantify extracellular phages, whereas a second aliquot was vortexed with chloroform before titration to quantify total (intracellular and extracellular) phages. Titrations were performed using the double-layer agar method, and all assays were conducted in triplicate.

### Phage genome sequencing, assembly, and annotation

High-molecular-weight genomic DNA was extracted from high-titer lysates of bacteriophages PTolC-28 and PTolC-69. For PTolC-28, whole-genome sequencing was performed using Single Molecule Real-Time (SMRT) sequencing technology on the PacBio Sequel platform (Pacific Biosciences). SMRTbell libraries were prepared using the DNA Template Prep Kit according to the manufacturer’s instructions, and genomic DNA was sheared to approximately 10 kb fragments using a Covaris g-TUBE. For PTolC-69, a hybrid sequencing strategy was employed, combining PacBio Sequel IIe long-read sequencing (SMRTbell prep kit 3.0) and Illumina short-read sequencing (150 bp paired-end reads), with Illumina data quality-filtered using fastp v0.23.0 (Chen et al., 2018). Library preparation and sequencing for both bacteriophages were performed by Azenta Life Sciences (Suzhou, China).

For PTolC-28, PacBio Sequel continuous long reads (subreads) were assembled *de novo* with Canu v1.7 (Koren et al., 2017). The draft contig was polished by iterative mapping of the long reads for error correction, yielding a single circular contig of 148,242 bp with an average sequencing depth of 93.2×. For PTolC-69, PacBio Sequel IIe HiFi reads were assembled de novo using Hifiasm v0.13-r308 (Cheng et al., 2021). The resulting long-read assembly was further corrected using Illumina short-read data and Pilon (Walker et al., 2014), producing a single contig of 87,728 bp (average depth: 1236×).

Both genomes were annotated with Pharokka v1.9.1 (Bouras et al., 2023). CDS were predicted with PHANOTATE v1.6.7 (McNair et al., 2019), tRNAs identified using tRNAscan-SE 2.0 (Chan et al., 2021), and PHROG functional categories assigned via MMseqs2 (Steinegger and Söding, 2017; Terzian et al., 2021). Antibiotic-resistance genes and virulence factors were screened using the CARD Resistance Gene Identifier (RGI; Alcock et al., 2023), considering only Perfect and Strict hits, and the VFDB VFanalyzer (Liu et al., 2019), respectively.

### Comparative genomic and phylogenetic analyses

Circular genome maps were generated with pyCirclize v1.10.1 (Shimoyama, 2022a), with CDS colored according to PHROG functional modules.

Genus- and species-level taxonomy was assigned with taxMyPhage v0.3.7 (Millard et al., 2025), using genome-wide intergenomic nucleotide similarity against the ICTV reference set. The two closest references per query were retained for synteny analysis. Query genome assemblies were reoriented to begin at the large terminase subunit (*terL*) gene using Dnaapler (Bouras et al., 2024). Selected reference genomes were reoriented to match the queries with a custom Biopython script (Cock et al., 2009). EasyFig-style synteny plots (Sullivan et al., 2011) were created with pyGenomeViz v1.6.1 (Shimoyama, 2022b). Adjacent genomes were compared using BLASTn (BLAST+ v2.17.0; Camacho et al., 2009; *e*-value threshold 1 × 10^−3^). Alignments of ≥200 bp and ≥70% identity were visualized as identity-shaded links.

TerL protein sequences of PTolC-28 (667 aa) and PTolC-69 (533 aa) were used as queries in BLASTp searches (BLAST+ v2.17.0) against the NCBI nr database, retaining one representative per organism. Because the two phages belong to different subfamilies, two 14-taxon datasets were constructed, Tree A (PTolC-28 with *Justusliebigvirus* members; outgroup *Klebsiella* phage vB_KpM_FBKp34) and Tree B (PTolC-69 with *Felixounavirus* members and the sister genera *Mooglevirus* and *Suspvirus*; outgroup *Erwinia* phage TP1 and phiEa104). Sequences were aligned with MAFFT v7.526 (--auto; Katoh and Standley, 2013) and trimmed using trimAl v1.5.1 (-gappyout; Capella-Gutiérrez et al., 2009; yielding 690 and 381 sites, respectively). Maximum-likelihood trees were inferred with IQ-TREE v3.1.2 (Minh et al., 2020) under ModelFinder-selected models (Kalyaanamoorthy et al., 2017; LG + R2 and LG + G4, respectively) with 1,000 ultrafast bootstrap (Hoang et al., 2018) and 1,000 SH-aLRT replicates. Trees were visualized with toytree v3.0.11 (Eaton, 2020); support is shown as SH-aLRT/UFBoot.

### Thermal and pH stability testing

The stability of phages PTolC-28 and PTolC-69 was tested using wild-type *E. coli* MG1655 as the indicator host. For thermal stability, phage suspensions (~1 × 10^8^ PFU/mL in LB) were incubated at 4, 25, 37, 45, 55, 65, or 75 °C. Aliquots taken at 1 h and 4 h were immediately chilled on ice and titred by the double-layer agar method. For pH stability, phage suspensions were prepared in LB adjusted to pH 2–12 in increments of one unit using HCl or NaOH, incubated at 37 °C, and titred at 1 h and 4 h. Conditions of 37 °C and pH 7 served as references. Titers are expressed as PFU/mL. The detection limits were 10^3^ PFU/mL (thermal) and 10^2^ PFU/mL (pH).

### Antimicrobial susceptibility testing

Minimum inhibitory concentrations (MICs) of 15 antibiotics against the *E. coli* isolates were determined by broth microdilution in sterile 96-well plates. Antibiotic stock solutions were serially diluted twofold in culture medium across plate wells. Overnight cultures in LB broth were diluted 1:100 into fresh LB medium and grown to mid-logarithmic phase (OD_600_ ≈ 0.5). These cultures were then further diluted 10,000-fold into wells, resulting in approximately 1 × 10^5^ CFU/mL in a final volume of 200 μL per well. Plates were incubated at 37 °C, and bacterial growth was kinetically monitored by measuring optical density at 600 nm (OD_600_) every 2 h for 16 h using a microplate reader. The MIC was operationally defined as the lowest antibiotic concentration yielding an OD_600_ below 0.1 after 16 h of incubation. MICs are reported as the modal values from three technical replicates across three independent biological replicates; per-replicate fold changes were calculated and summarized as geometric mean ± geometric standard deviation across biological replicates.

### Determination of phage host range

The host range of the purified bacteriophages was assessed against the 10 MDR *E. coli* isolates using a standard spot assay. Briefly, bacterial lawns of each isolate were prepared by the double-layer agar method. Aliquots (10 μL) of phage lysate (1 × 10^9^ PFU/mL) were spotted onto each lawn, followed by overnight incubation at 37 °C. Lytic activity was evaluated based on plaque clarity and scored using a four-point scale: +++ (complete lysis), ++ (slightly turbid lysis), + (weak lysis), and − (no lysis).

### PAS assay

The synergistic effect between phages and antibiotics was evaluated using a checkerboard assay in a 96-well plate format (Gu Liu et al., 2020; Yehia et al., 2025). Antibiotics were serially diluted along the x-axis, and phages were serially diluted along the y-axis. Each well was inoculated with a target *E. coli* strain at a final concentration of 1 × 10^6^ CFU. Phage lysates were added at final multiplicities of infection (MOIs) ranging from 10^1^ to 10^−5^, while antibiotics were applied in twofold serial dilutions. The total volume in each well was adjusted to 200 μL using fresh LB medium. Plates were incubated at 37 °C, and bacterial growth was monitored kinetically by measuring OD_600_. Growth suppression in each well was quantified as the ratio of endpoint OD_600_ values (at 16 h) to the untreated control wells (ΔOD/OD_control_), expressed as a percentage. Lower percentages indicated greater growth inhibition. The effective antibiotic concentration was defined as the lowest concentration achieving ≥80% growth suppression (residual growth ≤20% of control). The fold reduction was calculated as the ratio of this concentration with antibiotic alone versus antibiotic plus phage. The minimum effective phage titer was the lowest titer that achieved this reduction. Synergy was quantified using the Bliss independence model (Bliss, 1939). For each combination, the Bliss excess (observed minus expected fractional inhibition) was calculated from single-agent effects. Combinations with an excess > 0.1 were scored as synergistic.

### Animal infection model and *in vivo* efficacy evaluation

Thirty-two one-day-old female Huangma chicks were housed under controlled conditions (35 ± 1 °C, 65 ± 5% relative humidity) with continuous illumination, antibiotic-free starter feed, and water provided ad libitum. After 2 days of acclimatization, chicks were randomly assigned into four groups (*n* = 8 per group; Supplementary Table S2).

On day 3, all chicks were challenged via intramuscular injection into the thigh with 3 × 10^8^ CFU of the MDR strain GDW21C03 (in 0.1 mL PBS prepared from mid-log LB cultures). Three hours post-challenge, treatments were administered by oral gavage as described in Supplementary Table S2. In the combination treatment group, phage and antibiotic were delivered sequentially in separate gavages to avoid potential physicochemical incompatibility.

At 3 days post-challenge, surviving chicks were humanely euthanized. Spleen and bilateral lung tissues were aseptically collected, weighed, and homogenized in PBS (0.7 mL/g tissue) using 2-mm steel beads at 70 Hz (three cycles of 60 s with 5-s intervals). Serial dilutions of tissue homogenates were plated onto EMB agar and incubated overnight at 37 °C. Colonies displaying a characteristic metallic green sheen were enumerated, and bacterial loads were expressed as log_10_ CFU/g tissue.

Animal experiments were approved by the Committee on the Ethics of Animal Experiments of Huazhong Agricultural University (approval no. 202604110007) and conducted following institutional guidelines.

### Statistical analysis

All statistical analyses were performed using Python (version 3.11) with SciPy (version 1.15), statsmodels (version 0.14), and pingouin (version 0.5) libraries. Statistical tests were two-tailed, and *p*-values < 0.05 were considered statistically significant.

Gene expression analyses were performed on ΔCt values, which are approximately normally distributed, rather than on exponentially transformed 2^−ΔΔCt^ values (Livak and Schmittgen, 2001). Differences in *tolC* expression between each avian pathogenic *E. coli* isolate and the reference strain *E. coli* MG1655 were evaluated by one-way ANOVA followed by Dunnett’s *post hoc* test. Homogeneity of variances was confirmed using Levene’s test (*p* = 0.99). Results are reported as the mean ± standard deviation (SD) from three independent biological replicates.

For the chick infection model, bacterial load data were log_10_-transformed prior to analysis. Samples yielding no detectable colonies at the lowest dilution tested (10 μL undiluted homogenate; theoretical limit of detection [LOD] = 70 CFU/g tissue) were assigned a value of LOD/2 (35 CFU/g; log_10_ = 1.54) for statistical purposes. Data were analyzed by two-way ANOVA with treatment (four levels) and tissue (two levels) as between-subject factors, followed by Tukey’s honestly significant difference (HSD) *post hoc* test for all pairwise comparisons within each tissue. Levene’s test confirmed homogeneity of variances (*p* = 0.71). Because Shapiro–Wilk tests indicated deviations from normality in several groups, results were further confirmed by non-parametric Mann–Whitney U tests with Holm-Šídák correction, yielding consistent conclusions (all comparisons involving Combination treatment significant at *p* < 0.01). Effect sizes are reported as partial eta-squared (η^2^_p_) for ANOVA and Hedges’ g for pairwise comparisons.

## Results

### Isolation and characterization of MDR *Escherichia coli* from poultry

To investigate AMR among avian pathogenic *E. coli*, 10 strains were isolated from pathological samples (heart and liver) obtained from deceased chickens collected at poultry farms in Guangdong Province, China. Molecular typing revealed a genetically diverse bacterial population, characterized by multiple STs, serotypes, and phylotypes. Detailed strain characteristics are presented in Supplementary Table S3.

### Antimicrobial susceptibility profiles of *Escherichia coli* isolates

**Figure 1:**
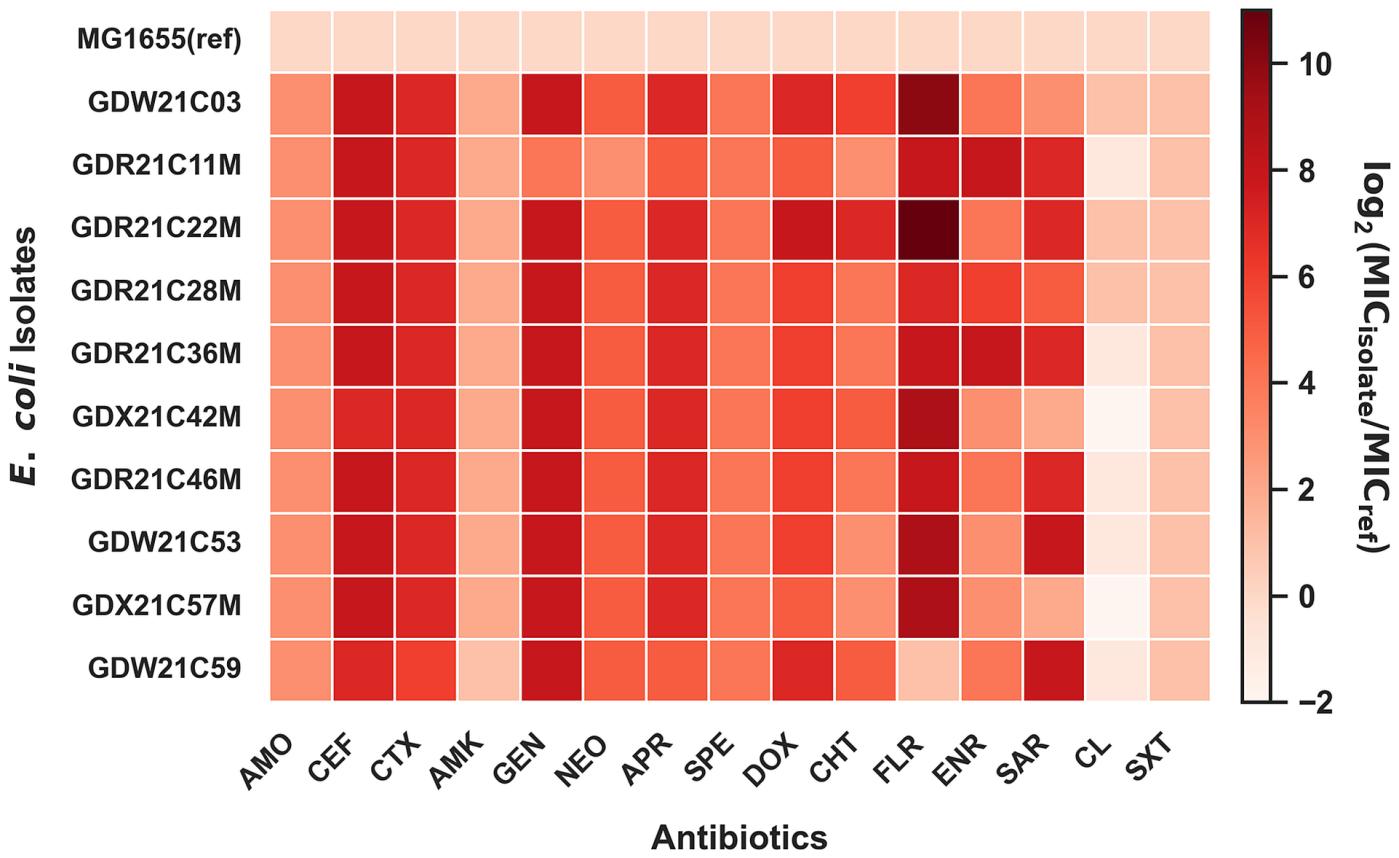
Antimicrobial resistance profiles of avian pathogenic *E. coli* isolates. Heatmap of MIC fold-changes for 15 antibiotics across the ten MDR *E. coli* isolates relative to the susceptible reference *E. coli* MG1655. Color intensity represents log_2_(MIC_isolate_/MIC_ref_); darker red indicates higher resistance, and lighter shades indicate susceptibility comparable to the reference. AMO, amoxicillin; CEF, ceftiofur; CTX, cefotaxime; AMK, amikacin; GEN, gentamicin; NEO, neomycin; APR, apramycin; SPE, spectinomycin; DOX, doxycycline; CHT, chlortetracycline; FLR, florfenicol; ENR, enrofloxacin; SAR, sarafloxacin; CL, colistin; SXT, trimethoprim-sulfamethoxazole. Detailed MIC fold-change values are provided in Supplementary Table S4.

The antimicrobial susceptibility of the 10 *E. coli* isolates was assessed by measuring MICs of 15 antibiotics from seven major drug classes, expressed as fold-changes relative to the susceptible reference strain, *E. coli* MG1655 (Figure 1). All isolates exhibited a pronounced and consistent MDR phenotype. Uniform high-level resistance was observed against β-lactams, with MIC fold-changes ranging from 64- to >128-fold for ceftiofur and exceeding 64-fold for cefotaxime compared to MG1655. Resistance within the tetracycline class showed MIC elevations ranging from 8- to >256-fold, and fluoroquinolone MICs increased by 4- to 512-fold. Among aminoglycosides, MICs for gentamicin, neomycin (NEO), apramycin, and spectinomycin were consistently elevated (≥8-fold). In contrast, amikacin (AMK) MICs showed only modest increases (2- to 4-fold), indicating retained susceptibility. This extensive, high-level resistance across multiple structurally distinct antibiotic classes confirmed the MDR classification for all 10 isolates. Detailed MIC fold-change data for each strain and antibiotic are summarized in Supplementary Table S4.

### Elevated expression of *tolC* in MDR isolates

Given the central role of TolC as a critical component of major efflux pumps (e.g., AcrAB-TolC) in MDR among *E. coli* isolates, we investigated whether elevated tolC expression correlated with the observed high-level antibiotic resistance. Basal *tolC* mRNA levels in all 10 clinical isolates were quantified by RT-qPCR relative to the antibiotic-susceptible reference strain, *E. coli* MG1655, under antibiotic-free, mid-logarithmic growth conditions, using 16S rRNA as the endogenous control.

**Figure 2:**
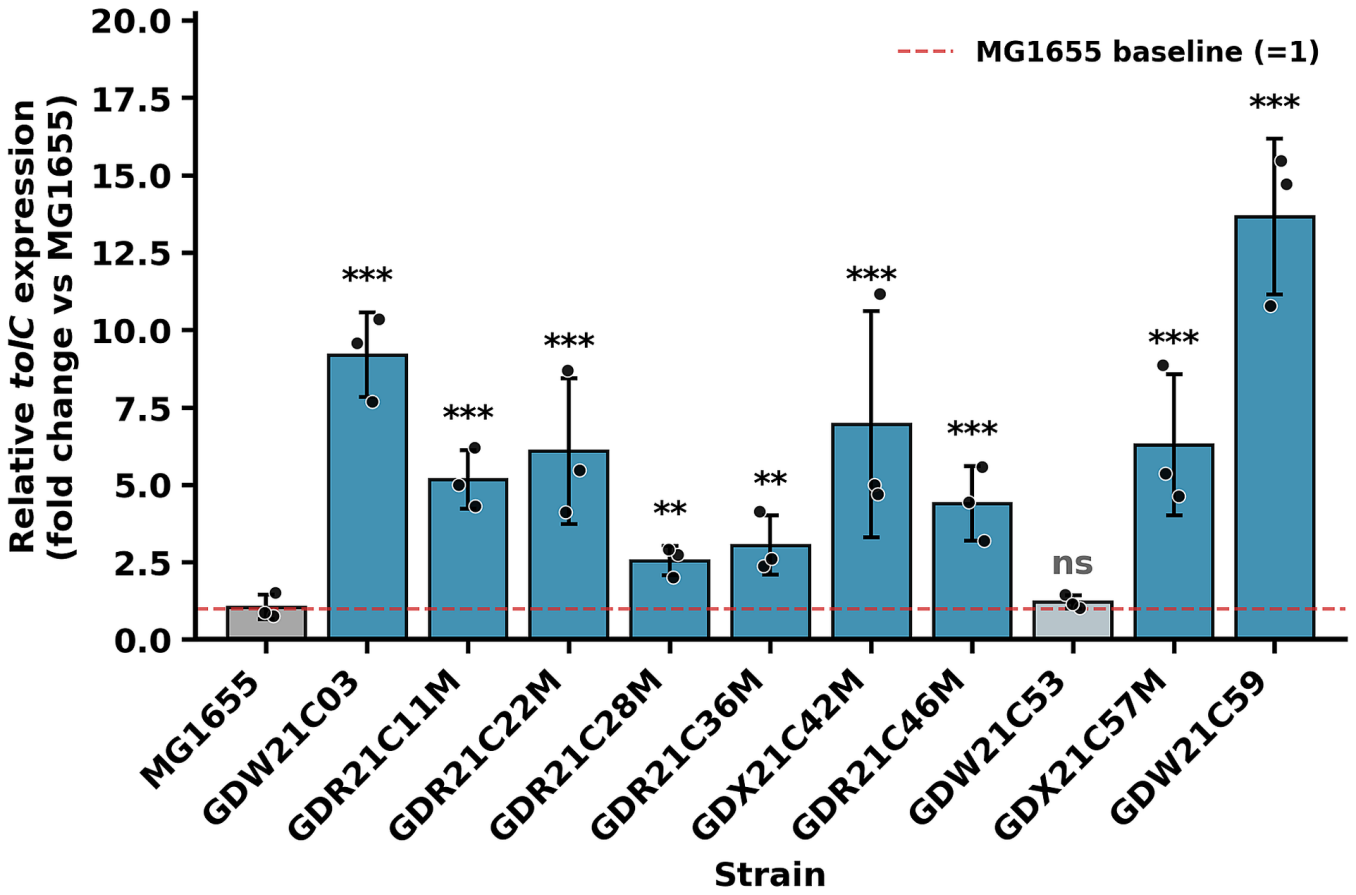
Basal *tolC* mRNA expression in multidrug-resistant isolates. Basal *tolC* mRNA expression in the 10 clinical isolates relative to the reference strain *E. coli* MG1655 was quantified by RT-qPCR, with 16S rRNA as the endogenous reference. Bars represent the mean fold-change relative to MG1655 from three independent biological replicates. Error bars indicate the standard deviation (SD) across biological replicates, and individual data points represent each biological replicate. Statistical significance was assessed by Dunnett’s test (*p < 0.05, **p < 0.01, ***p < 0.001; ns, not significant).

Nine of the ten MDR clinical isolates demonstrated significantly increased *tolC* mRNA levels compared to MG1655 (Dunnett’s test, all *p* < 0.01; Figure 2), with fold changes ranging approximately from 2.5- to 13.7-fold. Isolate C59 exhibited the highest *tolC* expression (~14-fold), followed by C03 (~9-fold), C42M (~7-fold), and C57M (~6-fold). Only one isolate (C53) displayed *tolC* expression comparable to MG1655 (~1.2-fold, *p* = 0.99). Although the magnitude of *tolC* upregulation did not precisely correlate with MIC fold changes for individual antibiotic classes, this is consistent with previous reports that efflux-mediated resistance involves multiple mechanisms rather than efflux alone (Nikaido and Pagès, 2012; Camp et al., 2021). The consistent upregulation of *tolC* observed across most isolates identifies *tolC* overexpression as a common molecular feature underlying the MDR phenotype in these poultry-derived *E. coli* strains. These findings provided a rationale for subsequently targeting phages that exploit TolC as a receptor.

### Targeted isolation and identification of TolC-dependent phages

**Figure 3:**
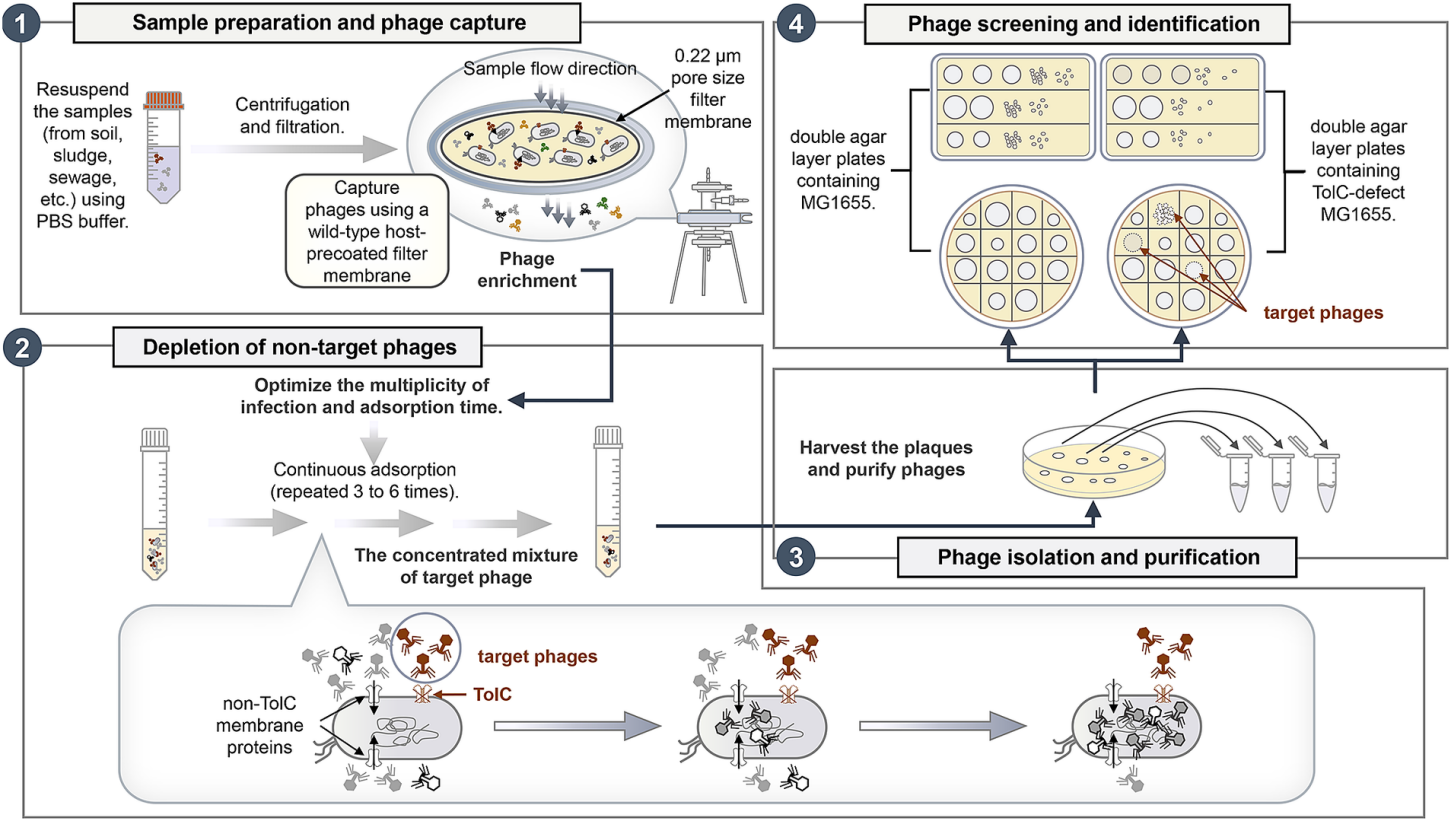
Schematic workflow of the sequential positive–negative selection strategy for the targeted isolation of TolC-dependent bacteriophages from environmental samples. (Step 1) Sample preparation and phage capture (positive selection). Environmental samples are resuspended in PBS, clarified by centrifugation, and passed through a filter membrane pre-coated with a wild-type *E. coli* MG1655 lawn. Non-adsorbing particles flow through. (Step 2) Depletion of non-target phages (negative selection). Captured phages are recovered into suspension and subjected to 3–6 rounds of pre-incubation with *E. coli* MG1655Δ*tolC*, followed by centrifugation to remove adsorbed phages. TolC-dependent phages (orange) remain in the supernatant and become progressively enriched. (Step 3) Phage isolation and purification. The TolC-enriched suspension is plated by the double-layer agar method on a wild-type MG1655 indicator lawn, and individual plaques are harvested. (Step 4) Phage screening and identification. Candidates are spotted in parallel onto wild-type MG1655 and MG1655Δ*tolC* lawns. Phages with clear lysis on the wild-type but impaired plaque formation on the Δ*tolC* lawn (orange arrows) are designated TolC-dependent and selected for three successive rounds of single-plaque purification.

To selectively isolate bacteriophages utilizing TolC as a receptor, we developed a novel targeted screening approach (summarized in Figure 3). The method involved sequential positive and negative selection steps. During positive selection, bacteria-free filtrates from poultry fecal samples were passed through membranes precoated with wild-type *E. coli* MG1655, enabling the capture of phages recognizing surface receptors, including TolC. Subsequently, in the negative selection step, the recovered phage suspension was pre-incubated with the MG1655*ΔtolC* mutant to deplete phages capable of infecting independently of TolC. To preserve natural phage diversity and avoid selection biases resulting from differential replication rates, we deliberately omitted conventional liquid enrichment, employing instead a direct-plating strategy.

This targeted procedure yielded several phage candidates, from which two distinct bacteriophages, designated PTolC-28 and PTolC-69, were isolated and selected for further characterization after three rounds of single-plaque purification.

### Phenotypic characterization of isolated TolC-dependent phages

**Figure 4:**
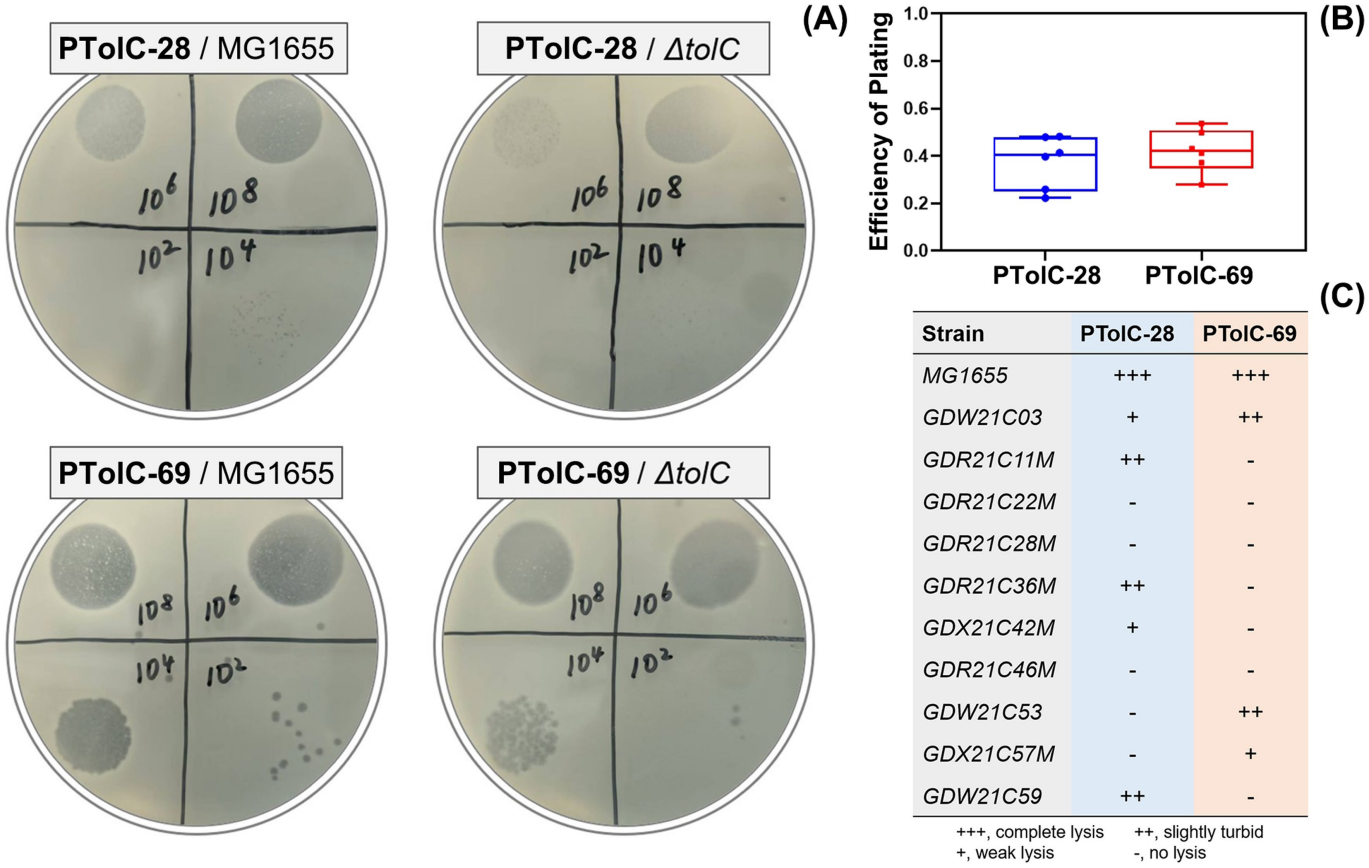
TolC-dependent infection and host range of phages PTolC-28 and PTolC-69. **(A)** Spot assay of TolC dependency on wild-type *E. coli* MG1655 (left) versus the isogenic Δ*tolC* mutant (right). Ten-fold serial dilutions of phage lysate (10^2^–10^8^ PFU/mL, 10 μL per spot) were applied to double-layer agar lawns. Top panels, PTolC-28; bottom panels, PTolC-69. Images are representative of at least two independent experiments. **(B)** Efficiency of plating (EOP = titer~Δ*tolC*~ / titer~WT~) of PTolC-28 (blue) and PTolC-69 (red) on the MG1655Δ*tolC* mutant relative to the wild-type host, determined by double-layer agar titration in parallel. Box plots show the median (center line), interquartile range (box), and minimum-to-maximum range (whiskers); individual points represent independent biological replicates (*n* = 6 per phage). **(C)** Host range against the 10 MDR avian *E. coli* isolates and the laboratory host *E. coli* MG1655, assessed by spot assay. Lytic activity was scored by clarity of the lysis zone: +++, complete lysis; ++, slightly turbid; +, weak lysis; −, no lysis. Blue and orange columns indicate scoring for PTolC-28 and PTolC-69, respectively. Results represent the consensus of at least two independent experiments.

To confirm their TolC receptor dependency, we assessed the infectivity of phages PTolC-28 and PTolC-69 using wild-type *E. coli* MG1655 and its isogenic *ΔtolC* mutant via spot and plaque assays (Figure 4A). Both phages produced large, clear plaques on the wild-type host but significantly smaller, more turbid plaques on the *ΔtolC* mutant, indicative of impaired infection. Quantitative analysis showed reduced plating efficiencies on the *ΔtolC* mutant to 37.6 ± 11.0% for PTolC-28 (range: 22.3–48.2%) and 42.2 ± 9.2% for PTolC-69 (range: 27.9–53.7%), compared to wild-type MG1655 (Figure 4B). This substantial reduction verified TolC as the primary receptor for both phages, although residual infectivity on the *ΔtolC* mutant suggested possible utilization of alternative receptors.

To evaluate their therapeutic potential, we determined the host ranges of both phages against the panel of ten MDR *E. coli* isolates described above (Figure 4C). Results showed that both phages lysed clinical isolates beyond their original propagation host (MG1655). Phage PTolC-28 exhibited a relatively broad host range, infecting 5 of the 10 MDR strains (GDW21C03, GDR21C11M, GDR21C36M, GDX21C42M, and GDW21C59). In contrast, PTolC-69 displayed a narrower host spectrum, lysing 3 isolates (GDW21C03, GDW21C53, and GDX21C57M). Collectively, these data indicate that the isolated phages, particularly PTolC-28, demonstrate promising lytic activity against diverse MDR avian pathogenic *E. coli*.

**Figure 5:**
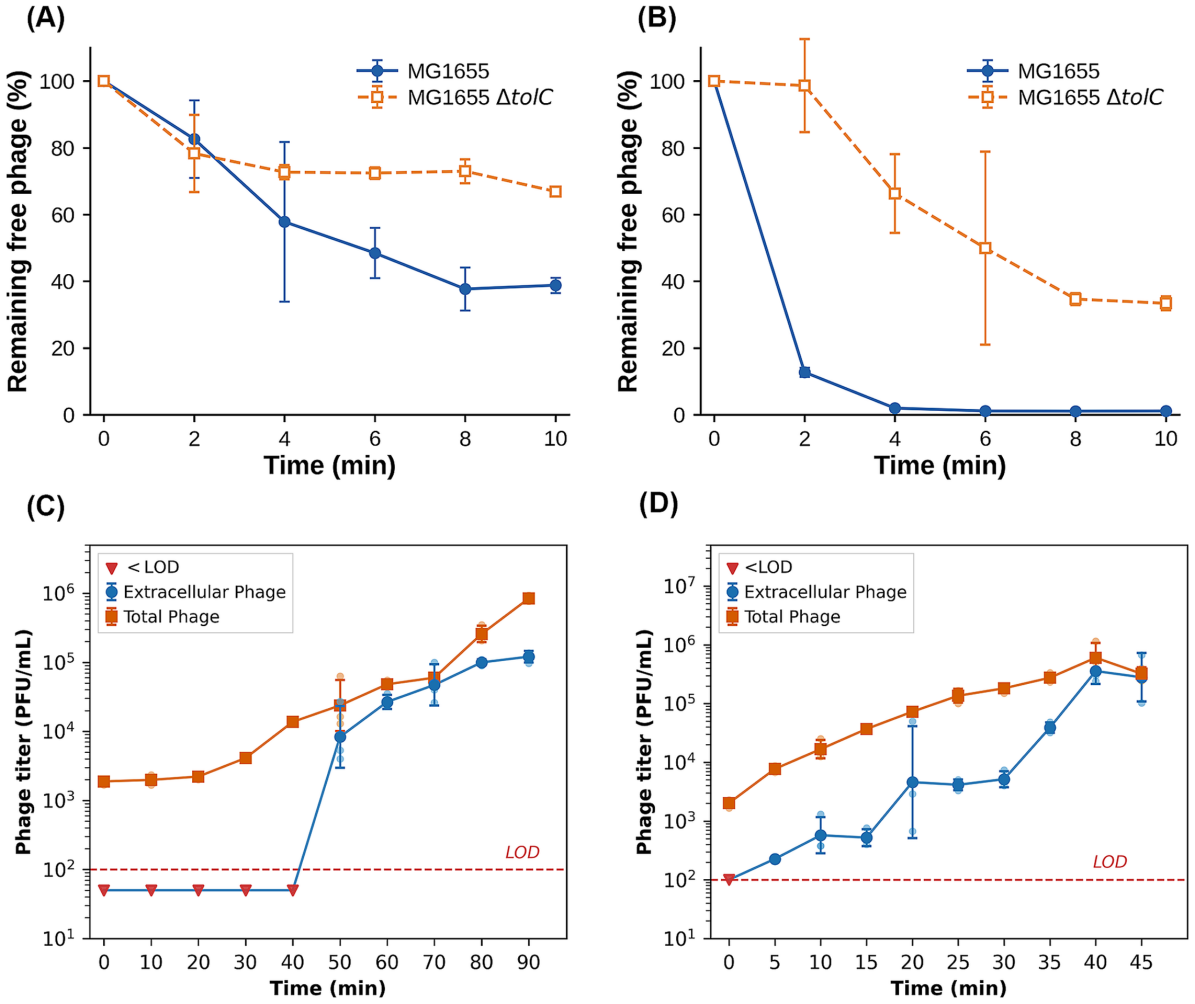
Adsorption kinetics and one-step growth of phages PTolC-28 and PTolC-69. **(A,B)** Adsorption of PTolC-28 **(A)** and PTolC-69 **(B)** to wild-type *E. coli* MG1655 (filled circles, solid line) and the isogenic Δ*tolC* mutant (open squares, dashed line); free phage remaining in the supernatant was titred at the indicated times and expressed as a percentage of the input titer at *t* = 0. **(C,D)** One-step growth curves of PTolC-28 **(C)** and PTolC-69 **(D)** on wild-type MG1655, showing extracellular (free) phage (circles) and total (intracellular plus extracellular) phage recovered after chloroform treatment (squares). Dashed lines mark the limit of detection (10^2^ PFU/mL); downward triangles denote values at or below it. Data are presented as the mean ± SD of three technical replicates.

Adsorption assays further confirmed receptor dependency during initial attachment. Both phages adsorbed effectively to the wild-type host but showed markedly slower and reduced adsorption on the Δ*tolC* mutant (Figures 5A,B). This was most notable for PTolC-69 (≥98% adsorbed within 4 min on wild-type vs. ~67% after 10 min on Δ*tolC*). PTolC-28 adsorbed more slowly (~61% by 8–10 min). The residual TolC-independent adsorption matched partial plating efficiency on the Δ*tolC* mutant (Figure 4B), suggesting secondary receptors. One-step growth assays (Figures 5C,D) showed PTolC-28 had an eclipse period of ~30 min, latent period of ~40–50 min, and burst size of approximately 450 PFU/cell, with progeny plateauing at 80–90 min. PTolC-69 had a more rapid replication cycle; free phage levels increased significantly by ~15 min, plateaued at ~3 × 10^5^ PFU/mL by ~40 min, and had a burst size of ~250 PFU/cell.

**Figure 6:**
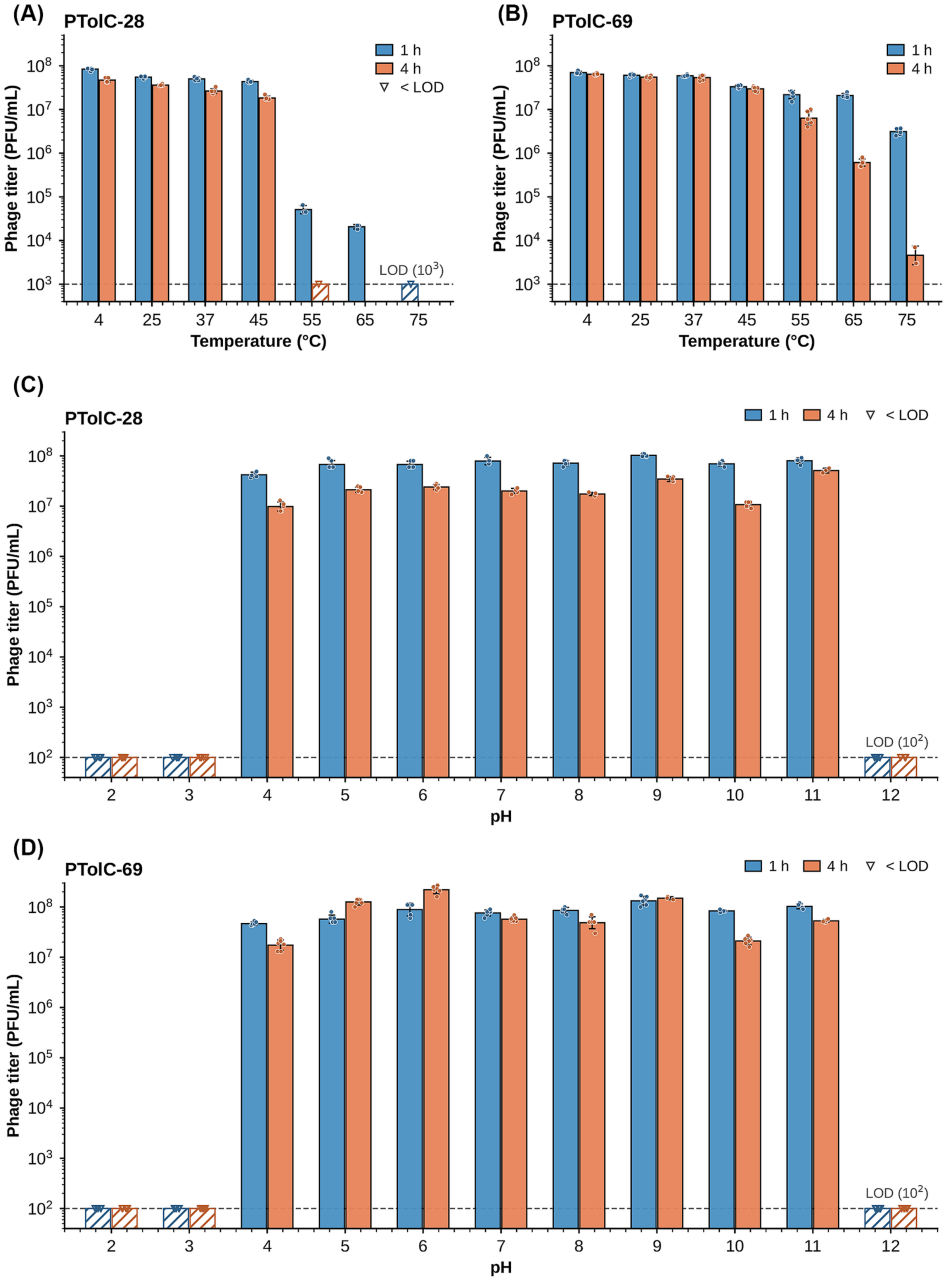
Stability of phages PTolC-28 and PTolC-69 to temperature and pH. Phages were exposed to the indicated temperatures (4–75 °C, **A,B**) or pH values (pH 2–12; **C,D**) for 1 h (blue) or 4 h (orange), and surviving titers were determined by plaque assay. Panels show PTolC-28 **(A,C)** and PTolC-69 **(B,D)**. Bars are geometric mean titers (PFU/mL, log scale) with individual technical replicates overlaid as symbols; error bars indicate ± 1 SD (log₁₀ scale). Dashed lines mark the limit of detection (10^3^ PFU/mL for temperature, 10^2^ PFU/mL for pH); hatched open bars with a downward triangle denote titers that fell below the limit of detection.

Finally, stability testing (Figure 6) evaluated conditions relevant for storage and application. Both phages were stable across a wide pH range, retaining titers of ~10^7^–10^8^ PFU/mL between pH 4 and pH 11 after 1 and 4 h at 37 °C. Titers fell below detection limits only under extreme conditions (pH ≤ 3 and pH 12) (Figures 6C,D). The phages differed significantly in thermal tolerance (Figures 6A,B). PTolC-28 remained infectious from 4 to 45 °C but was rapidly inactivated at ≥55 °C. In contrast, PTolC-69 remained infectious up to 65 °C and was still detectable at 75 °C. This difference increased with prolonged incubation, as PTolC-69 retained substantial titers at 55–65 °C over 4 h, conditions that completely inactivated PTolC-28. Both phages thus tolerate typical physiological and production pH ranges, with the greater thermal stability of PTolC-69 being advantageous for downstream processing and field deployment.

### Genomic characterization of PTolC-28 and PTolC-69

**Figure 7:**
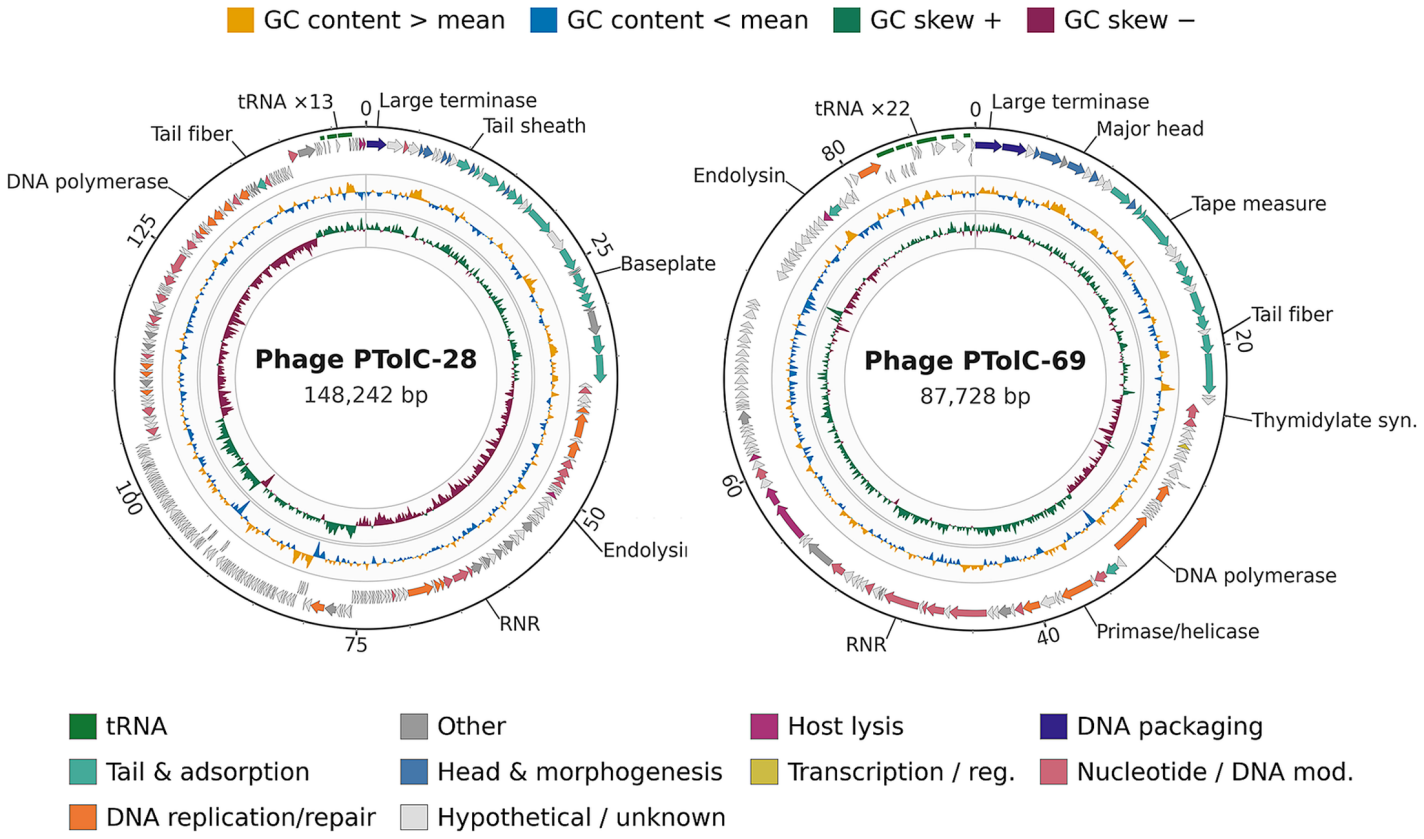
Circular genome maps of *Escherichia coli* phages vB_EcoM_PTolC28 (PTolC-28, left) and vB_EcoM_PTolC69 (PTolC-69, right). Both genomes begin (position 0) at the large terminase gene. Rings (outer → inner): position scale (kb); tRNA genes; forward- and reverse-strand CDS (arrows colored by PHROG functional module, see key); GC content as deviation from the genome mean (orange, above; blue, below); GC skew [(G − C)/(G + C); green, positive; dark red, negative]. Selected hallmark genes are labeled. Per-CDS module assignments are in Supplementary Table S5.

Annotation confirmed distinct genome architectures and supported biosafety assessment (Figure 7). PTolC-28 (148,242 bp; GC content 37.48%; coding density 93.7%) encoded 281 CDS and 13 tRNA genes; 82 CDS (29.2%) were assigned functional categories, while 199 encoded hypothetical proteins. PTolC-69 (87,728 bp; GC content 39.0%; coding density 91.1%) encoded 148 CDS and 22 tRNA genes; 45 CDS (30.4%) were functionally annotated, and 103 were hypothetical, typical for phage genomes.

Both genomes encoded hallmark genes for structural, packaging, and DNA-metabolism, including large terminase, head/capsid proteins, tail proteins, DNA polymerase, endolysin, and ribonucleotide reductase. Consistent with a strictly lytic lifecycle, neither genome encoded CDS related to integration, excision, or lysogeny. No antibiotic-resistance genes were detected by RGI, and no toxins, secretion systems, or dedicated virulence determinants were identified by VFanalyzer. PTolC-28 contained three ORFs encoding enzymes for the conserved dTDP-L-rhamnose biosynthesis pathway (*rmlA*, *rmlC*), widespread in bacteria and plants (Giraud and Naismith, 2000). As biosynthetic enzymes rather than virulence factors, these do not represent transferable virulence determinants. PTolC-69 returned no VFDB matches. The absence of resistance and lysogeny genes supports further evaluation as therapeutic candidates. Full annotations are provided in Supplementary Tables S5, S6.

Genome-wide intergenomic similarity analysis assigned the phages to distinct genera (Supplementary Table S7). For PTolC-28, all 10 closest references belonged to the genus *Justusliebigvirus* (92.0–95.3% similarity). Its top hit, *Escherichia* phage alia (95.25%), exceeded the ICTV species threshold, placing PTolC-28 within the species *Justusliebigvirus alia*. PTolC-69’s closest references belonged to genus *Felixounavirus* (91.1–93.3% similarity); its top hit, *Escherichia* phage CL1 (OK040806; 93.30%), remained below the species threshold, indicating PTolC-69 likely represents a novel species.

**Figure 8:**
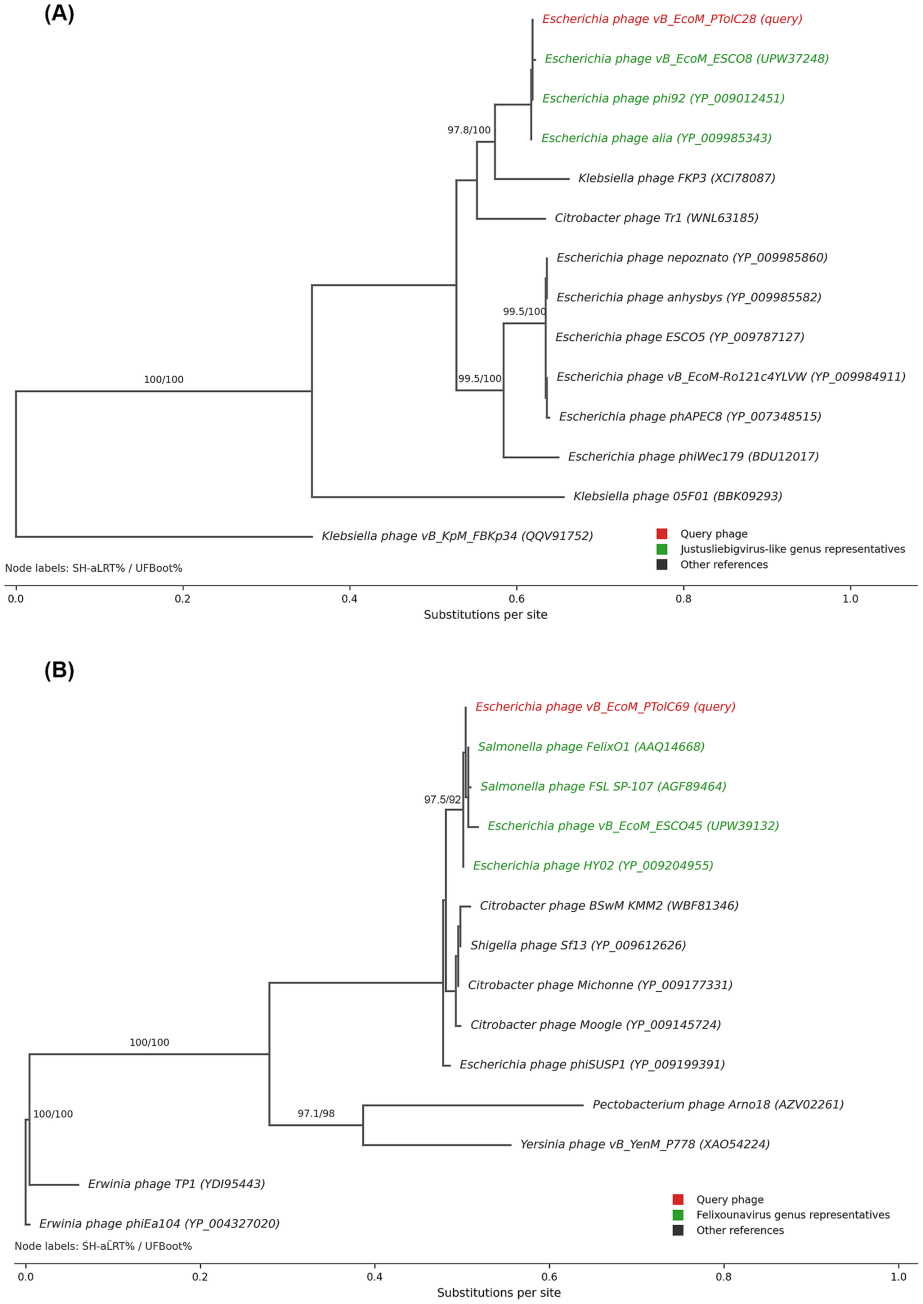
Maximum-likelihood TerL phylogenies. **(A)** PTolC-28 within the genus *Justusliebigvirus* (subfamily *Stephanstirmvirinae*), rooted on *Klebsiella* phage vB_KpM_FBKp34. **(B)** PTolC-69 within the genus *Felixounavirus* (subfamily *Ounavirinae*), rooted on the *Erwinia* phage outgroup (TP1 and phiEa104). Trees were inferred from large terminase (TerL) amino-acid sequences. Query phages, red; genus members, green; other reference taxa, gray. Branch lengths are to scale (substitutions per site); node support is shown as SH-aLRT (%)/ultrafast bootstrap (%).

Maximum-likelihood phylogenies based on the TerL protein supported these genus-level assignments (Figure 8). In Tree A (Figure 8A), PTolC-28 clustered within the *Justusliebigvirus* radiation, specifically in a *Justusliebigvirus*-like, phage alia-related TerL context, with strong support at the relevant node (SH-aLRT/UFBoot = 97.8/100). The tree was rooted at the midpoint of the *Klebsiella* phage vB_KpM_FBKp34 outgroup branch. In Tree B (Figure 8B), PTolC-69 grouped among *Felixounavirus* members (*Salmonella* phage FelixO1, *Escherichia* phage HY02, *Escherichia* phage vB_EcoM_ESCO45, and *Salmonella* phage FSL_SP-107). PTolC-69 was clearly separated from the sister genera *Mooglevirus* and *Suspvirus* and from the *Erwinia* phage outgroup (TP1 and phiEa104). The relevant genus-level placement node was strongly supported (SH-aLRT/UFBoot = 100/100). Shallow internal nodes among closely related sequences were poorly resolved, as expected from a single conserved marker gene. These did not affect genus-level placement and were not further interpreted.

**Figure 9:**
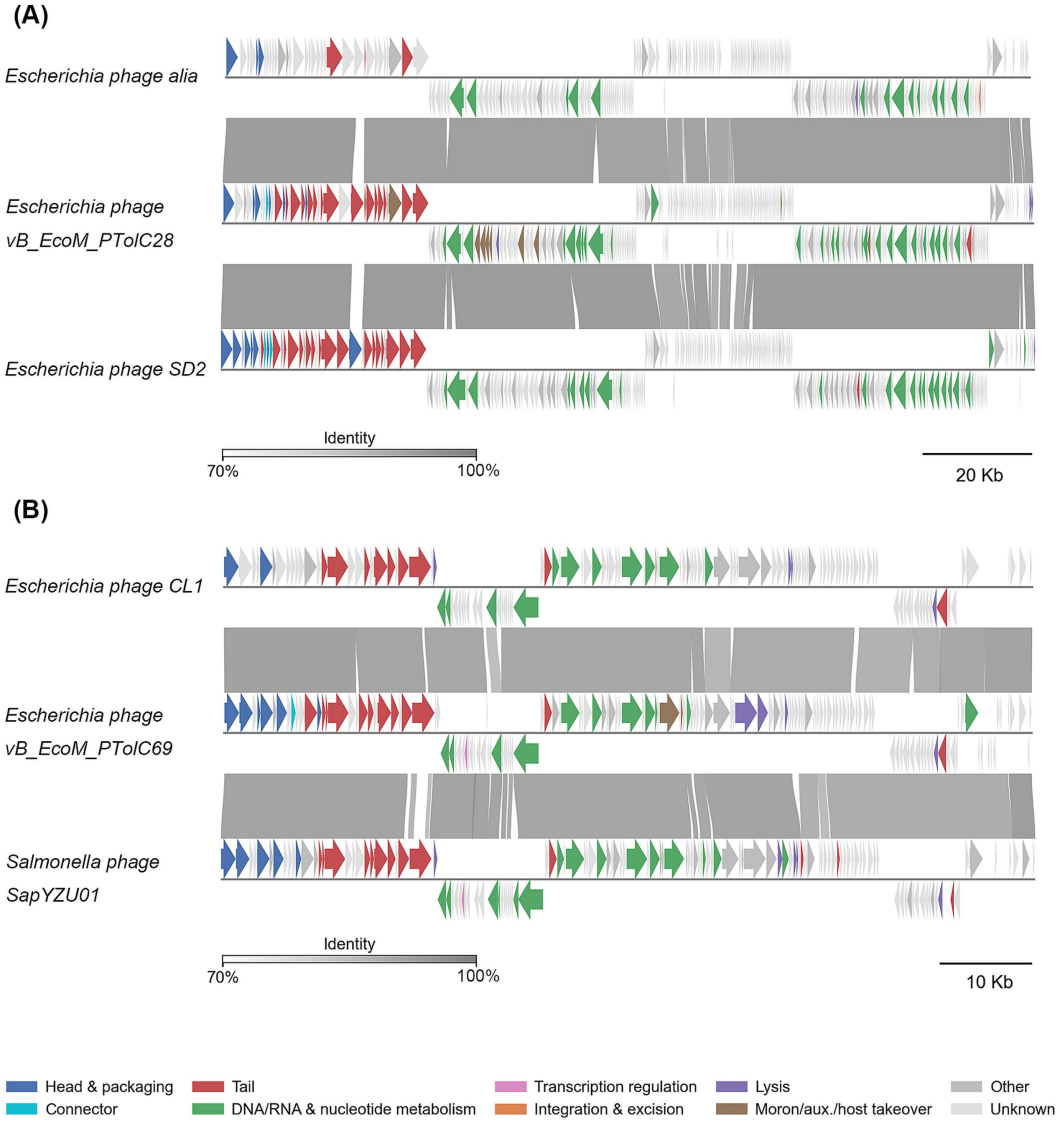
Whole-genome synteny of PTolC-28 and PTolC-69 with their two closest reference phages, ordered by decreasing taxMyPhage similarity. **(A)** PTolC-28 with Escherichia phage alia (MN850632) and Escherichia phage SD2 (PQ821640). **(B)** PTolC-69 with Escherichia phage CL1 (OK040806) and Salmonella phage SapYZU01 (MT409176). Arrows show CDS position and orientation, colored by functional category; gray links show BLASTn similarity (≥200 bp, ≥70% identity) between adjacent tracks, shaded by nucleotide identity. The functional-category color key is common to both panels. Scale bars, 20 kb **(A)** and 10 kb **(B)**.

Whole-genome synteny comparison reinforced these affiliations (Figure 9). Each query phage showed high collinearity with its two closest classified relatives: PTolC-28 with *Escherichia* phages alia (MN850632) and SD2 (PQ821640) (Figure 9A), and PTolC-69 with *Escherichia* phage CL1 (OK040806) and *Salmonella* phage SapYZU01 (MT409176) (Figure 9B). BLASTn homology covered structural, packaging, and DNA-metabolism modules, with nucleotide identities of 79.8–100.0% for PTolC-28 and 87.0–98.6% for PTolC-69. Gene order was conserved between queries and references, consistent with previous taxonomic and phylogenetic assignments. Concordance among three independent analyses, intergenomic similarity, TerL phylogeny, and genome-wide synteny, confirmed genus-level placement of both phages.

### Evolutionary trade-off: PTolC-28 reverses antibiotic resistance

To determine whether selection by PTolC-28 imposes a trade-off between phage resistance and antibiotic susceptibility, five PTolC-28-susceptible MDR isolates (GDW21C03, GDR21C11M, GDR21C36M, GDX21C42M, and GDW21C59) were subjected to co-culture selection at MOI ≥ 10,000, followed by single-colony purification and five serial passages in phage-free medium to exclude transient phenotypes. Under these conditions, stable phage-resistant derivatives were recovered from only two strains (GDW21C03 and GDR21C11M), whereas no stable resistant colonies emerged from the remaining three strains (GDR21C36M, GDX21C42M, and GDW21C59) within the 48-h recovery window despite repeated attempts. This limited emergence of resistance suggests that evolutionary escape under PTolC-28 pressure is constrained in most tested genetic backgrounds. Among the two resistant strains, only the GDW21C03-derived mutant (designated TrPTolC28-C03) exhibited coordinated *tolC* suppression and multi-class antibiotic resensitization, consistent with an evolutionary trade-off.

**Figure 10:**
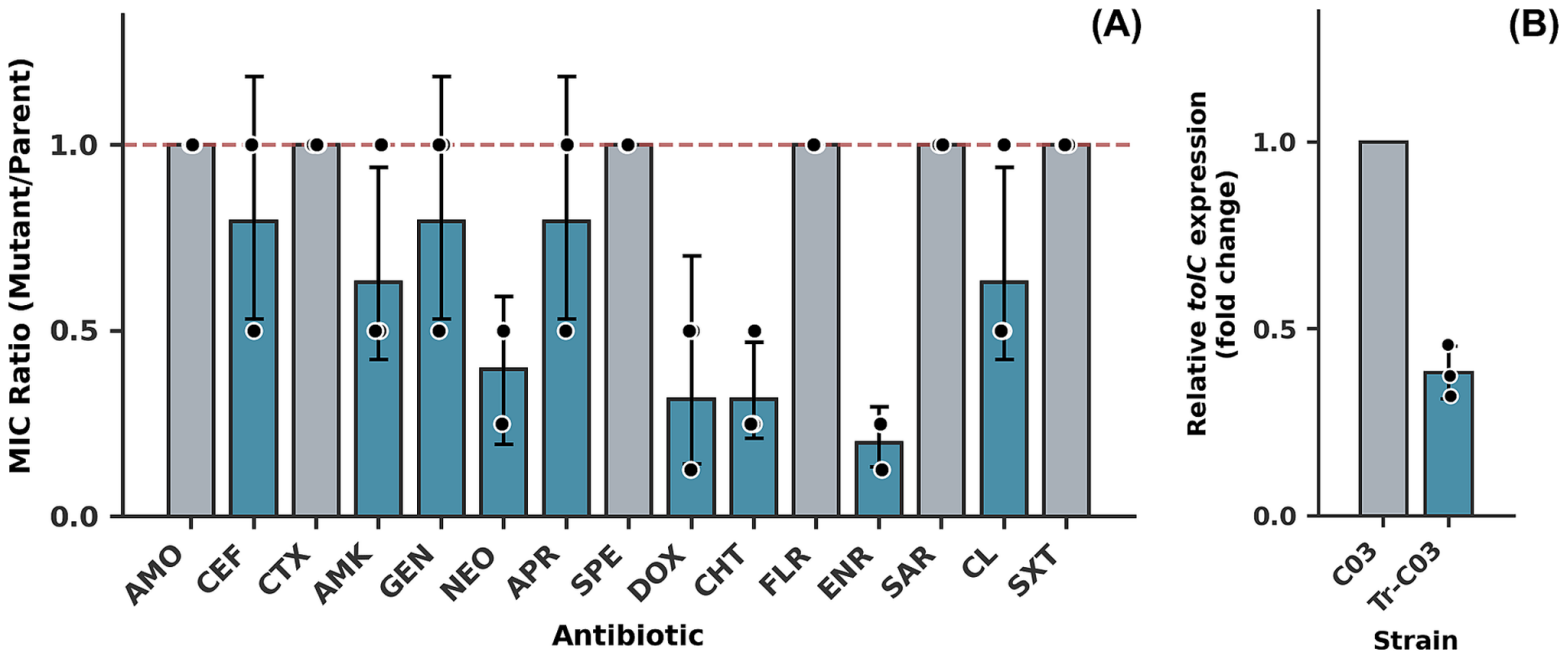
PTolC-28 selection drives transcriptional suppression of *tolC* and collateral resensitization to multiple antibiotic classes in the multidrug-resistant strain GDW21C03. **(A)** Antimicrobial susceptibility of the phage-resistant mutant TrPTolC28-C03 relative to its parental strain GDW21C03, expressed as the MIC ratio (MIC _mutant_ / MIC _parent_) for 15 antibiotics. Bars represent the geometric mean from three independent biological replicates; error bars indicate the geometric standard deviation (SD). The horizontal dashed line at $y = 1$ represents no change in susceptibility, whereas values $ < 1$ indicate collateral resensitization. Antibiotic abbreviations are consistent with those defined in Figure 1. **(B)** Relative *tolC* mRNA expression in TrPTolC28-C03 (Tr-C03) compared with parental GDW21C03 (C03), quantified by RT-qPCR with 16S rRNA as the endogenous reference and the parental strain as the calibrator (relative expression = 1). Bars represent the mean fold-change from three independent biological replicates; error bars, SD.

The phage-resistant mutant TrPTolC28-C03 was first evaluated for *tolC* expression. Relative *tolC* mRNA levels were reduced by more than 60% compared with the parental strain (Figure 10B). Sanger sequencing of the *tolC* coding region revealed no mutations relative to the parent, indicating that the observed downregulation is not attributable to coding sequence changes. This transcriptional alteration was accompanied by marked resensitization to multiple antibiotics. Antimicrobial susceptibility testing showed collateral sensitization relative to the parental GDW21C03 strain (Figure 10A). Using a conservative threshold of ≥2-fold MIC reduction to account for technical variability inherent to broth microdilution, four antibiotics across three mechanistically distinct classes demonstrated biologically meaningful resensitization; all are known substrates of the TolC-dependent efflux system(s). The most pronounced effect was observed for enrofloxacin (ENR; MIC reduced to 18.8 ± 6.3% of the parental value, ~5.3-fold), followed by chlortetracycline (CHT; 33.3 ± 14.4%, ~3-fold), doxycycline (DOX; 37.5 ± 21.7%, ~2.7-fold), and NEO (50.0 ± 25.0%, 2-fold). MICs for the remaining antibiotics either fell within the ±1 two-fold dilution range considered technically indistinguishable or showed no reduction. These results indicate that selection by PTolC-28 can reverse MDR in *E. coli* by favoring mutants with reduced efflux pump expression.

In contrast, the phage-resistant derivative from GDR21C11M maintained *tolC* transcript levels comparable to those of the parental strain, and targeted MIC testing against representative AcrAB-TolC substrates showed no corresponding pattern of collateral resensitization, indicating that resistance arose via a route that largely preserved efflux function. Together with the absence of stable resistant mutants in the other three susceptible strains, these findings identify GDW21C03 as uniquely susceptible to phage-induced transcriptional suppression of *tolC* under PTolC-28 selection. More broadly, the divergent outcomes across the five backgrounds, trade-off (C03), efflux-preserving escape (C11M), and no accessible escape (C36M, C42M, C59), indicate that the evolutionary consequences of TolC-targeted phage selection depend on strain-specific regulatory architecture and the spectrum of available escape routes, consistent with the host-dependent mutational landscape reported for TolC-targeting phages (Burmeister et al., 2020).

### Synergistic action: PTolC-69 potentiates antibiotic efficacy

**Figure 11:**
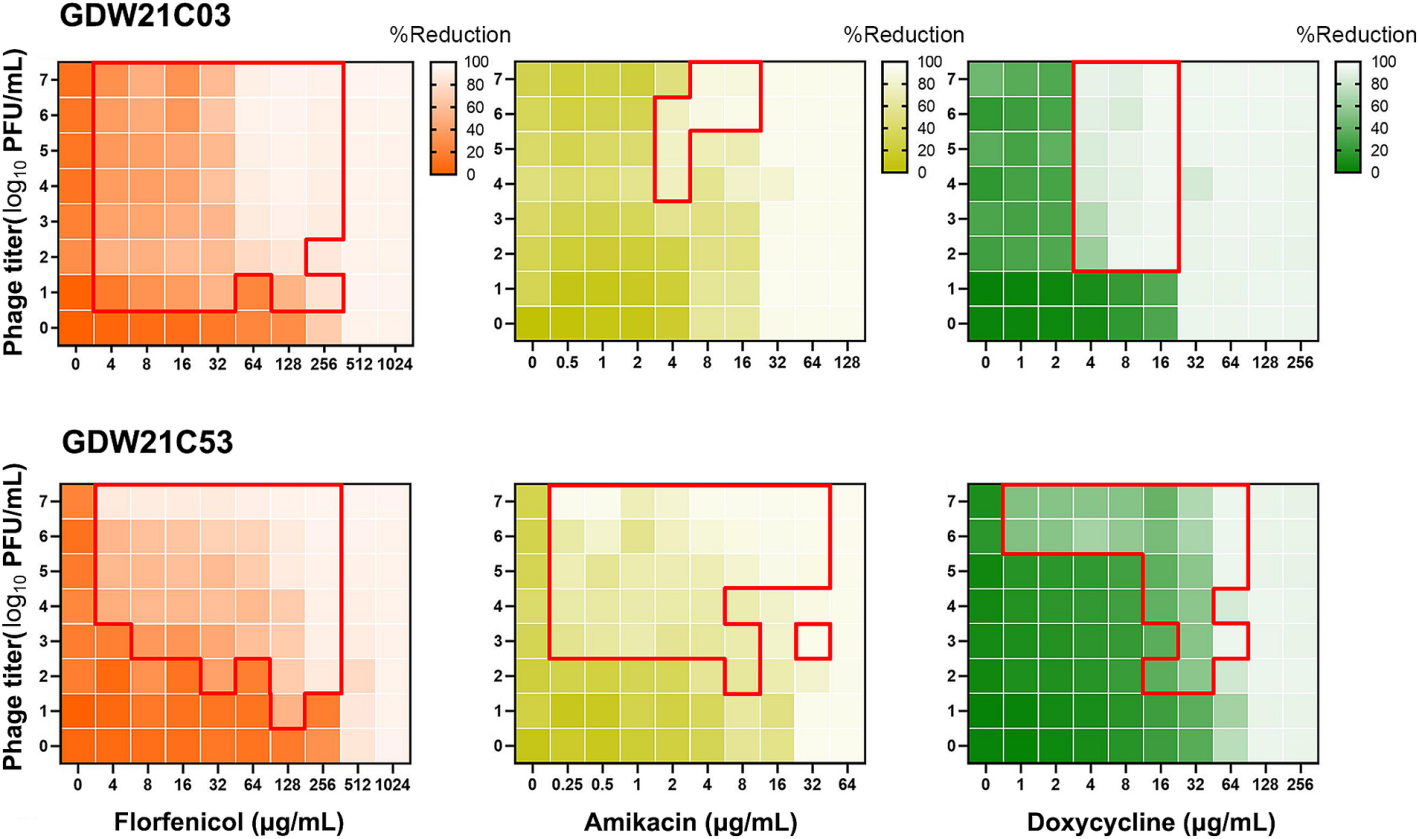
*In vitro* phage-antibiotic synergy (PAS) between PTolC-69 and conventional antibiotics against multidrug-resistant *E. coli*. Checkerboard analysis of PAS *in vitro*. PTolC-69 was combined with florfenicol (left, orange), amikacin (center, yellow), or doxycycline (right, green) against MDR strains GDW21C03 (top row) and GDW21C53 (bottom row). x-axis, antibiotic concentration (μg/mL, two-fold serial dilutions); *y*-axis, phage titer (log₁₀ PFU/mL). Color intensity represents residual growth at the 16-h endpoint (endpoint OD₆₀₀ / control OD₆₀₀ × 100%), with darker shading indicating greater suppression (see color bars). Red outlines mark combinations with a Bliss independence excess > 0.1, denoting synergy. Data represent the mean of three independent biological replicates.

Unlike the evolutionary trade-off observed with PTolC-28, co-evolution experiments involving phage PTolC-69 did not consistently yield antibiotic resensitization. Therefore, we investigated an alternative therapeutic potential, PAS. The combined inhibitory effects of PTolC-69 and three antibiotics (DOX, AMK, and FLR) were evaluated against MDR strains GDW21C03 and GDW21C53 using checkerboard assays (Figure 11).

These assays demonstrated synergistic interactions between PTolC-69 and multiple antibiotics against both bacterial strains, with the strongest synergy observed in GDW21C03. Growth suppression was quantified by comparing endpoint OD_600_ values to untreated control wells, expressed as a percentage, where lower percentages indicate greater suppression.

Phage-antibiotic interactions were evaluated using the Bliss independence model. Combinations with a Bliss excess greater than 0.1 were classified as synergistic (red outlines, Figure 11). PTolC-69 exhibited synergy with all three antibiotics, observed in five of six phage-antibiotic-strain combinations. Florfenicol produced the most consistent synergy (maximum Bliss excess: 0.59 [GDW21C03], 0.68 [GDW21C53]; 67 and 54% synergistic combinations, respectively). Doxycycline also synergized with PTolC-69 (0.68 [GDW21C03], 0.44 [GDW21C53]). Amikacin synergized strongly against GDW21C53 (0.69; 53%) but showed predominantly additive effects with local antagonism against GDW21C03 (maximum Bliss excess: 0.30; 10% synergistic combinations). Thus, PTolC-69-mediated PAS was both strain- and antibiotic-specific.

Against GDW21C03, PTolC-69 reduced effective concentrations of DOX (8-fold: 32 → 4 μg/mL at 10^4^ PFU/mL) and FLR (8-fold: 512 → 64 μg/mL at 10^3^ PFU/mL). Against GDW21C53, DOX and FLR showed moderate synergy, with reductions of 2-fold (128 → 64 μg/mL, from 10^2^ PFU/mL) and 4-fold (512 → 128 μg/mL, from 10^4^ PFU/mL), respectively. AMK achieved the greatest reduction (8-fold, 32 → 4 μg/mL at 10^6^ PFU/mL), but only at high phage titers. Conversely, AMK against GDW21C03 showed only a 4-fold reduction (32 → 8 μg/mL) at 10^6^ PFU/mL, with no effect at lower titers, consistent with its largely additive Bliss profile. Therefore, the strongest potentiation at low phage titers occurred with DOX and FLR against GDW21C03, while AMK synergy was robust only for GDW21C53 at higher titers, highlighting the strain- and antibiotic-specific nature of PTolC-69-mediated potentiation.

Collectively, these findings demonstrate that PTolC-69 enhances the efficacy of multiple antibiotic classes via PAS, representing a distinct therapeutic approach compared to the resistance reversal strategy observed with PTolC-28.

### *In vivo* synergistic efficacy of PTolC-69 and DOX in a chick infection model

To assess the clinical relevance of the observed phage–antibiotic synergy, we evaluated the combined efficacy of PTolC-69 and DOX in a chick infection model using the MDR *E. coli* strain GDW21C03. Treatment was initiated 3 h post-bacterial challenge, and chicks were assigned to four groups (*n* = 8 per group): untreated control, phage monotherapy, antibiotic monotherapy, and combination therapy. All birds survived the three-day post-challenge observation period, after which bacterial burdens in lung and spleen tissues were quantified.

**Figure 12:**
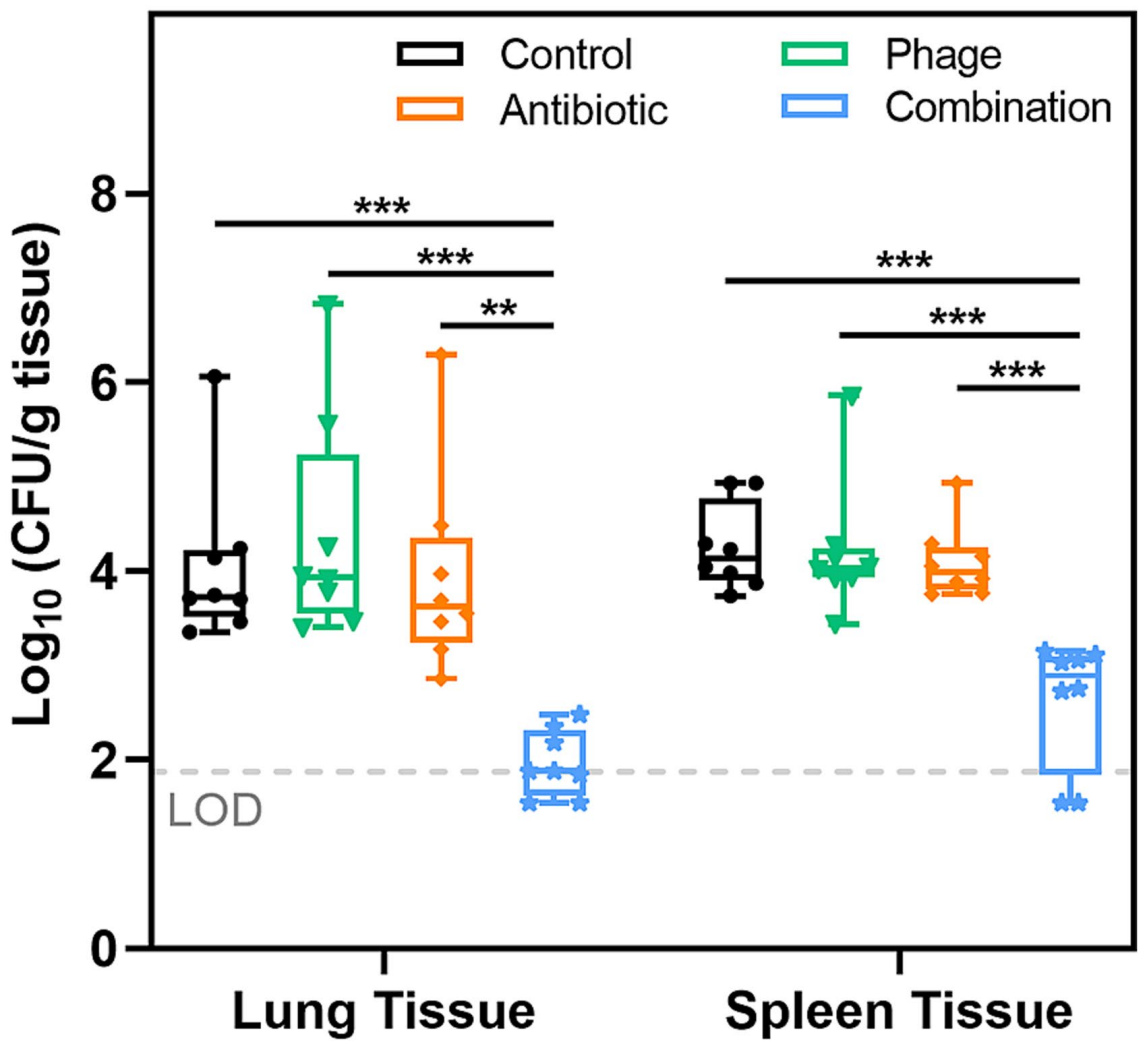
*In vivo* efficacy of PTolC-69 combined with doxycycline against MDR *E. coli* infection in a chick model. Bacterial loads in lung and spleen of chicks at 3 days post-challenge with GDW21C03, following treatment with PBS (Control), PTolC-69 alone (Phage), doxycycline alone (Antibiotic), or PTolC-69 plus doxycycline (Combination); *n* = 8 per group. Box plots show the median (center line), interquartile range (box), and minimum-to-maximum range (whiskers); individual data points are overlaid. The dashed line indicates the limit of detection (LOD = 70 CFU/g); samples below LOD were imputed as LOD/2 for statistical analysis. Statistical significance was determined by two-way ANOVA with Tukey’s *post-hoc* test (**p < 0.01, ***p < 0.001); only significant comparisons are shown.

Two-way ANOVA revealed a significant main effect of treatment on bacterial load (F_3_, _56_ = 23.84, *p* < 0.001, η^2^*p* = 0.56), with no significant effect of tissue type (*p* = 0.29) or treatment-by-tissue interaction (*p* = 0.50), indicating consistent treatment effects across both tissues (Figure 12). Tukey’s HSD *post hoc* comparisons demonstrated that neither phage nor antibiotic monotherapy significantly reduced bacterial burdens compared with untreated controls in either tissue (all *p* > 0.75). In contrast, combination therapy significantly reduced median bacterial loads by approximately 1.9 log_10_ CFU/g in lung and 1.6 log_10_ CFU/g in spleen relative to untreated controls, with several animals approaching or reaching the detection limit. Pairwise comparisons between the combination treatment and each of the three other groups were significant in both tissues (*p* < 0.001 for five of six comparisons; *p* = 0.001 for combination versus antibiotic treatment in lung), with large effect sizes (Hedges’ g range: −2.17 to −2.99). These results confirm that PTolC-69 and DOX act synergistically *in vivo* to suppress systemic MDR *E. coli* infections, consistent with the *in vitro* checkerboard assay findings.

## Discussion

In this study, we report the targeted isolation and characterization of two novel TolC-dependent bacteriophages, PTolC-28 and PTolC-69, from poultry farm environments, demonstrating their distinct yet complementary abilities to combat MDR in avian pathogenic *E. coli*. By employing a specifically designed positive–negative selection strategy, we successfully enriched TolC-dependent phages from complex environmental samples and confirmed their therapeutic relevance against a panel of MDR clinical isolates. Critically, our findings indicate that TolC-dependent phages exert antibacterial effects via two distinct mechanisms: PTolC-28 imposes an evolutionary trade-off that drives collateral resensitization to multiple antibiotics in permissive bacterial hosts (e.g., MDR strain GDW21C03), whereas PTolC-69 synergizes with conventional antibiotics to achieve potent bactericidal activity both *in vitro* and *in vivo*.

Our results confirm and extend the steering paradigm established for the OprM efflux channel in *P. aeruginosa* (Chan et al., 2016, 2018; Gurney et al., 2020; Kortright et al., 2022) and for *Burkholderia* (Ruest et al., 2023) to a new host species in an applied veterinary context. AcrAB-TolC and MexAB-OprM are counterpart RND systems within the outer membrane factor family. In addition to confirming this principle for *E. coli* TolC, our study introduces four elements: a prospective positive–negative scheme for receptor-directed phage isolation; identification of two distinct therapeutic modalities (evolutionary steering and PAS) from a single environmental niche; a novel escape route involving transcriptional downregulation of *tolC*, rather than efflux-preserving loop mutations seen with model phage TLS (Tamer et al., 2021); and *in vivo* proof-of-concept in a poultry infection model.

A central methodological contribution of this study is the establishment of a sequential positive–negative selection approach designed to enrich TolC-dependent bacteriophages directly from complex environmental samples. By intentionally omitting conventional liquid enrichment, our strategy maintains the inherent diversity of environmental phage communities, increasing the likelihood of isolating receptor-specific phages that might otherwise be overshadowed by fast-replicating, dominant populations (Twest and Kropinski, 2009; Hyman, 2019). Conceptually, this transforms what is traditionally a downstream confirmatory assay, differential plating on wild-type versus Δ*tolC* lawns, into an active, upstream selection procedure, enabling targeted, prospective isolation of receptor-specific bacteriophages. The successful isolation of PTolC-28 and PTolC-69 demonstrates the effectiveness of this approach and highlights its potential as a broadly applicable framework for selectively recovering bacteriophages targeting other therapeutically relevant bacterial surface structures. Genomic analyses further corroborated these findings. PTolC-28 belongs to the genus Justusliebigvirus (subfamily Stephanstirmvirinae) and is closely related to Escherichia phage phi92, a known TolC-utilizing member of the same genus (Burmeister et al., 2020; Maffei et al., 2021; Burmeister et al., 2023), supporting its TolC dependency. In contrast, PTolC-69 (87,728 bp; GC content 39.0%) belongs to the genus *Felixounavirus* (subfamily *Ounavirinae*), a lineage not previously recognized to contain TolC-dependent members. Recovering two TolC-dependent phages from distinct genera within the same environmental sample underscores the versatility of our positive–negative selection method.

The most clinically significant finding in this study is the evolutionary trade-off imposed by phage PTolC-28 on the MDR strain GDW21C03, wherein acquisition of phage resistance coincided with significant downregulation of *tolC* expression and collateral resensitization to four of the 15 antibiotics tested. This outcome aligns with the theoretical framework proposed by Chan et al., suggesting that bacteriophages targeting efflux pump components can induce bacterial populations into a fitness trade-off: preserving the receptor maintains antibiotic resistance but leads to phage susceptibility, whereas receptor downregulation or loss confers phage resistance at the cost of impaired drug efflux (Chan et al., 2016; Burmeister et al., 2020). The resistant mutant derived from GDW21C03 (TrPTolC28-C03) exhibited a greater than 60% reduction in *tolC* mRNA levels compared with its parental strain, despite no experimental selection for antibiotic sensitivity. This strongly suggests that phage selection alone was sufficient to induce functionally significant suppression of efflux pump activity. Sanger sequencing of the *tolC* coding region revealed no mutations in TrPTolC28-C03, indicating that transcriptional suppression likely results from upstream regulatory alterations involving known regulators of the *acrAB-tolC* operon. Importantly, the observed collateral resensitization pattern was mechanistically coherent with *tolC* downregulation: the four antibiotics exhibiting ≥2-fold MIC reductions belong to distinct chemical classes yet are all known substrates of TolC-dependent efflux systems, DOX, CHT, and ENR for AcrAB-TolC (Elkins and Nikaido, 2002; Baucheron et al., 2004; Venter et al., 2015) and NEO for AcrD-TolC (Rosenberg et al., 2000; Yamamoto et al., 2016), which shares TolC as its outer membrane channel with AcrAB. Conversely, antibiotics whose resistance is minimally reliant on TolC-mediated efflux (amoxicillin, cefotaxime, and trimethoprim-sulfamethoxazole) exhibited no MIC changes in TrPTolC28-C03. This substrate-specific distinction confirms that the phage-imposed suppression of *tolC* directly compromises TolC-dependent efflux rather than inducing generalized fitness costs associated with phage resistance.

However, this clear mechanistic trade-off observed in GDW21C03 was not replicated in other susceptible isolates. Three of the five susceptible strains (GDR21C36M, GDX21C42M, and GDW21C59) failed to yield stable phage-resistant mutants under our experimental conditions, and the single resistant mutant recovered from GDR21C11M maintained *tolC* expression levels and did not exhibit comparable collateral resensitization. This variability mirrors observations by Burmeister et al., who demonstrated that evolutionary outcomes following selection by TolC-targeting phages depend strongly on bacterial genetic background (Burmeister et al., 2020). We propose three strain-specific factors contributing to this observed heterogeneity. First, alternative resistance mechanisms, such as lipopolysaccharide (LPS) modifications, differ substantially between strains; those capable of readily employing LPS-based escape mechanisms may acquire phage resistance without affecting TolC function, as documented previously for phage U136B (Burmeister et al., 2020). Second, *tolC* expression is regulated by multiple layers of control, involving transcriptional activators (MarA, SoxS, Rob), indirect repressors (AcrR), and post-transcriptional regulators (SdsR) (Zhang et al., 2008; Wright et al., 2025); existing variation at these regulatory nodes may influence whether phage-imposed selection pressures primarily affect *tolC* expression or alternative targets. Third, the relative contribution of AcrAB-TolC efflux to antibiotic resistance varies across clinical isolates (Camp et al., 2021); strains harboring additional plasmid-encoded resistance determinants likely face fewer constraints on efflux-mediated resistance (Cunrath et al., 2019). Thus, the evolutionary trajectories induced by TolC-targeted phage selection are shaped by the interplay among receptor accessibility, regulatory flexibility, and the availability of redundant resistance mechanisms within each bacterial genetic context.

Our findings also invite comparison with the mutational landscape previously characterized for the laboratory model phage TLS, extensively used to explore evolutionary plasticity in TolC. In that system, resistance typically arises from mutations in extracellular loops of TolC, which preserve efflux function, thus creating an “evolutionarily decoupled” scenario where phage resistance and antibiotic resistance are separable at the protein level (Tamer et al., 2021). In contrast, the GDW21C03-derived mutant in our study (TrPTolC28-C03) acquired phage resistance without mutations in the *tolC* coding sequence, instead exhibiting a greater than 60% reduction in *tolC* transcript abundance. This finding indicates that the evolutionary pathway in this strain operates primarily at the transcriptional level rather than through coding sequence alterations. This distinction has significant mechanistic implications: transcriptional suppression of *tolC* inherently compromises the entire AcrAB-TolC efflux system, whereas loop mutations that preserve protein sequence generally maintain efflux functionality. This transcriptional mechanism likely explains the pronounced, coordinated resensitization observed in TrPTolC28-C03 across three distinct antibiotic classes, all established substrates of TolC-dependent efflux. The current data cannot determine whether this transcriptional adaptation arises intrinsically from PTolC-28 targeting epitopes intolerant to single-residue substitutions, thus directing evolutionary responses toward regulatory modulation, or whether it results from unique regulatory features in GDW21C03. Resolving these possibilities will require whole-genome sequencing of resistant mutants and structural characterization of the PTolC-28-TolC interaction, both of which represent priorities for future investigations. Similarly informative would be the genomic analysis of the phage-resistant mutant derived from strain GDR21C11M, which retained efflux function and may share mutational characteristics with the LPS-modification resistance pathway documented previously for other TolC-targeting phages.

PTolC-69 functions via a mechanistically distinct pathway from PTolC-28. Rather than imposing an evolutionary trade-off, PTolC-69 potentiated conventional antibiotics, with synergy confirmed by checkerboard assays and validated *in vivo*. This potentiation was strain- and antibiotic-specific: DOX and FLR showed synergy with PTolC-69 against both strains, whereas AMK synergized against GDW21C53 but was largely additive, with localized antagonism, against GDW21C03. A plausible explanation for this heterogeneity is that potentiation occurs through distinct mechanisms depending on drug class. DOX and FLR target the ribosome and are substrates of the constitutively expressed AcrAB-TolC pump (Elkins and Nikaido, 2002; Baucheron et al., 2004), making their intracellular accumulation efflux-dependent. Phage disruption of the shared TolC channel would directly relieve efflux, increasing their effective intracellular concentrations, consistent with observed synergy across strains. Aminoglycosides can also be extruded by the RND pump AcrD. However, TolC disruption alone does not significantly enhance aminoglycoside susceptibility in *E. coli* (Rosenberg et al., 2000). This indicates that intracellular aminoglycoside accumulation is primarily driven by proton-motive-force-dependent uptake rather than periplasmic efflux (Taber et al., 1987). Thus, TolC disruption would affect AMK efflux less directly than it affects DOX and FLR efflux. Instead, AMK potentiation may arise from phage-induced changes in membrane energization and metabolic state (Allison et al., 2011), increasing aminoglycoside uptake. Such effects would likely vary with host genetic background, explaining the strain-specific AMK synergy observed here. This hypothesis can be experimentally tested by evaluating PTolC-69 with aminoglycosides under controlled metabolic and energization conditions across additional strains. Finally, PTolC-69 potentiated all three antibiotics despite minimal intrinsic bactericidal activity (Figure 11). This indicates that phages do not need to be effective monotherapeutics to serve as potent antibiotic adjuvants, broadening the selection of phages suitable for combination therapy.

The *in vivo* findings require careful interpretation. Neither DOX nor PTolC-69 monotherapy significantly reduced bacterial burdens compared to untreated controls, a result mechanistically consistent with the characteristics of both the challenge strain and treatment agents. Specifically, GDW21C03 exhibited high-level DOX resistance (>64-fold MIC elevation relative to MG1655; Figure 1), and PTolC-69 monotherapy likely encountered pharmacokinetic limitations, potentially restricting effective phage exposure in infected tissues under our experimental dosing conditions (Dąbrowska, 2019). Despite these monotherapy limitations, the combination therapy produced approximately 80-fold (1.9 log_10_ CFU/g) and 40-fold (1.6 log_10_ CFU/g) reductions in bacterial loads within the lung and spleen, respectively. Such reductions align with magnitudes previously reported as clinically meaningful bacterial control in murine and avian phage therapy studies (Huff et al., 2003; Gencay et al., 2024), although complete bacterial clearance was not achieved and detectable bacteria persisted in some animals at the endpoint. The absence of significant main effects for tissue type or treatment-by-tissue interaction (*p* = 0.29 and *p* = 0.50, respectively) indicates comparable synergistic efficacy in both anatomical compartments assessed, without apparent organ-specific restrictions at this dosing schedule and timeframe. Collectively, these data provide proof-of-concept that PTolC-69-DOX combination therapy can effectively suppress systemic MDR *E. coli* infection under conditions in which each agent alone is ineffective. However, future studies should evaluate whether dose optimization, repeated dosing, or alternative administration routes can achieve complete bacterial eradication.

Several limitations of this study merit acknowledgment. First, the residual plating efficiency of approximately 38–42% on the MG1655Δ*tolC* mutant indicates that TolC is the primary but not the exclusive receptor for PTolC-28 and PTolC-69 (residual adsorption to the Δ*tolC* mutant; Figures 5A,B). The existence of secondary receptors could, in principle, allow bacteria to evolve phage resistance through alternative receptor modifications that preserve efflux function (Tamer et al., 2021), potentially diminishing the evolutionary trade-off under more complex *in vivo* or environmental conditions. Within our experimental framework, however, the marked collateral resensitization observed in the TrPTolC28-C03 mutant suggests that alternative receptor modifications did not substantially weaken the selective pressure targeting TolC. Nonetheless, the relative contributions of primary and secondary receptors to resistance evolution *in vivo* remain to be elucidated.

Second, while the chick infection model employed here establishes proof-of-concept for *in vivo* efficacy, it differs from typical poultry farming conditions in several important aspects. The intramuscular challenge route induces acute systemic infections, which may not accurately replicate intestinal or respiratory infections more commonly encountered in poultry production environments (Dziva and Stevens, 2008). Additionally, the use of neonatal chicks (3–6 days old) with immature immune systems might not reflect the immunological dynamics of infections occurring in older birds within commercial flocks. Validation of this combination therapy approach in models that more closely resemble natural field conditions will therefore be essential for practical implementation.

Whole-genome sequencing of the TrPTolC28-C03 mutant would facilitate identification of specific mutations within regulatory loci such as *acrR*, *marA*, or *soxS*, thereby clarifying the molecular mechanisms underlying the transcriptional suppression of *tolC* and determining whether the evolutionary trade-off occurs through defined, predictable genetic pathways. Structural characterization of the PTolC-28-TolC interaction, alongside evaluation of additional phage-host pairs, would help determine whether the observed bias toward transcriptional escape reflects an intrinsic property of PTolC-28 or is influenced by strain-specific factors, thereby establishing the broader applicability and constraints of this evolutionary trade-off strategy. Finally, comparative genomic analysis of PTolC-69 with other *Felixounavirus* phages, particularly focusing on tail fiber and baseplate structures, could identify key TolC-binding determinants and inform rational development of phage cocktails that combine multiple receptor-targeting mechanisms.

## Data Availability

The genome sequences generated for this study have been deposited in the National Center for Biotechnology Information (NCBI) under BioProject accession PRJNA1455400. The BioSample accessions are SAMN57364342 (Escherichia phage vB_EcoM_PTolC28) and SAMN57404621 (Escherichia phage vB_EcoM_PTolC69).
