# Supplementary material for: Targeted isolation of TolC-dependent phages reveals dual strategies for combating multidrug-resistant avian Escherichia coli: from evolutionary trade-offs to antibiotic synergy
Source: Front Microbiol. 2026 Jul 14;17:1868543. doi: 10.3389/fmicb.2026.1868543 (PMC13407771; doi:10.3389/fmicb.2026.1868543)
Supplement: Supplementary file 2 [file Table_1.DOCX]

**Supplementary Table S1.** Primers used in the tolC expression measurement

| Primer | Sequence |
| --- | --- |
| 16s-qPCR-F1 | ACGCAGGCGGTTTGTTAAGTCAGATGT |
| 16s-qPCR-R1 | TTCGCCACCGGTATTCCTCCAGATCT |
| tolC-qPCR-F1 | ACCTATAGCAACGGCTACCGCGAC |
| tolC-qPCR-R1 | ATCCCTGCTGCTTTTTCCTGCAGC |
| 16sRNA-qPCR-probe1 | TCCCCGGGCTCAACCTGGGAACTGCATCTGAT |
| tolC-qPCR-probe1 | ACGGCATCAACTCTAACGCGACCAGTGCGTCC |

**Supplementary Table S2.** Experimental Group Design for the In Vivo Chick Infection Model

| **Group** | **PBS(mL)** | **Phage Dose (PFU/bird)** | **Phage Volume (mL)** | **Antibiotic Dose (mg/bird)** | **Antibiotic Volume (mL)** | **Total Gavage Volume (mL)** |
| --- | --- | --- | --- | --- | --- | --- |
| Control | 1.5 | - | - | - | - | 1.5 |
| Phage | 0.5 | 1×10⁶ | 1.0 | - | - | 1.5 |
| Antibiotic | 1.0 | - | - | 2 | 0.5 | 1.5 |
| Combination |  | 1×10⁶ | 1.0 | 2 | 0.5 | 1.5 |

All groups: n = 8 birds per group. Phage: PTolC-69. Antibiotic: doxycycline. Combination groups received sequential separate gavages of phage followed immediately by antibiotic to avoid physicochemical incompatibility.

**Supplementary Table S3.** The isolated antibiotic-resistant strains used in this study

| Strains | MLST | Serotype | Phylotype | Source |
| --- | --- | --- | --- | --- |
| GDW21C03 | ST-7127 | H45 | A | Luoding, Guangdong Province |
| GDR21C11M | ST-2325 | O21:H25 | A | Yingde, Guangdong Province |
| GDR21C22M | ST-746 | H37 | A | Yingde, Guangdong Province |
| GDR21C28M | ST-3856 | O91:H25 | A | Yingde, Guangdong Province |
| GDR21C36M | ST-2614 | O25:H28 | A | Yingde, Guangdong Province |
| GDX21C42M | ST-155 | O116:H9 | A | Luoding, Guangdong Province |
| GDR21C46M | ST-398 | O39:H16 | A | Yingde, Guangdong Province |
| GDW21C53 | ST-7588 | H37 | A | Luoding, Guangdong Province |
| GDX21C57M | ST-218 | O103:H43 | A | Luoding, Guangdong Province |
| GDW21C59 | ST-616 | H21 | B1 | Luoding, Guangdong Province |

**Supplementary Table S4.** Results of the antibiotic resistance test

| Strains | Antibiotics concentration (μg/mL)*^a^* | | | | | | | | | | | | | | | | | | |
| --- | --- | --- | --- | --- | --- | --- | --- | --- | --- | --- | --- | --- | --- | --- | --- | --- | --- | --- | --- |
|  | β-Lactams | | | Aminoglycosides | | | | Tetracyclines | | | Chloramphenicol | | Fluoroquinolones | | |  | |  | |
|  | AMO | CEF | CTX | AMK | GEN | NEO | APR | SPE | DOX | CHT | FLR | ENR | | SAR | CL | | SXT | |  |
| MG1655 | 32 | <1 | <1 | 8 | 1 | 8 | 2 | 16 | 0.5 | 2 | 1 | 0.125 | | 0.125 | 1 | | 64 | |  |
| GDW21C03 | >128 | >128 | >64 | 32 | >128 | >128 | >128 | >128 | 64 | 128 | 1024 | 2 | | 1 | 2 | | >64 | |  |
| GDR21C11M | >128 | >128 | >64 | 32 | 16 | 64 | 64 | >128 | 16 | 16 | 256 | 32 | | 16 | 0.5 | | >64 | |  |
| GDR21C22M | >128 | >128 | >64 | 32 | >128 | >128 | >128 | >128 | 128 | >128 | 2048 | 2 | | 16 | 2 | | >64 | |  |
| GDR21C28M | >128 | >128 | >64 | 32 | >128 | >128 | >128 | >128 | 32 | 32 | 128 | 8 | | 4 | 2 | | >64 | |  |
| GDR21C36M | >128 | >128 | >64 | 32 | >128 | >128 | >128 | >128 | 32 | 32 | 256 | 32 | | 16 | 0.5 | | >64 | |  |
| GDX21C42M | >128 | 128 | >64 | 32 | >128 | >128 | >128 | >128 | 32 | 64 | 512 | 1 | | 0.5 | 0.25 | | >64 | |  |
| GDR21C46M | >128 | >128 | >64 | 32 | >128 | >128 | >128 | >128 | 32 | 32 | 256 | 2 | | 16 | 0.5 | | >64 | |  |
| GDW21C53 | >128 | >128 | >64 | 32 | >128 | >128 | >128 | >128 | 16 | 32 | 512 | 1 | | 64 | 0.5 | | >64 | |  |
| GDX21C57M | >128 | >128 | >64 | 32 | >128 | >128 | >128 | >128 | 16 | 16 | 512 | 1 | | 0.5 | 0.25 | | >64 | |  |
| GDW21C59 | >128 | 64 | 64 | 16 | >128 | >128 | 64 | >128 | 64 | 64 | 2 | 8 | | 64 | 0.5 | | >64 | |  |

*^a^*AMO: amoxicillin; CEF: ceftiofur; CTX: cefotaxime; AMK: amikacin; GEN: gentamicin; NEO: neomycin; APR: apramycin; SPE: spectinomycin; DOX: doxycycline;

CHT: chlortetracycline; FLR: florfenicol; ENR: enrofloxacin; SAR: sarafloxacin; CL: colistin; SXT: trimethoprim-sulfamethoxazole.
